# Mechanobiology-instructed biomaterials for osteoarthritis: Bridging mechanotransduction and disease modification

**DOI:** 10.1016/j.mtbio.2026.103819

**Published:** 2026-11-06

**Authors:** Jingxin Pan, Xi Wang, Pan Li, Jianbing Ma, Zhiyuan Wang, Zhao Zhang

**Affiliations:** a Department of Joint Surgery, Honghui Hospital, Xi'an Jiaotong University, Xi'an, 710054, China; b School of Medicine, Northwest University, Xi'an, Shaanxi, China; c Department of Orthopaedics, Shaanxi Provincial People's Hospital, Xi'an, Shaanxi, China; d Key Laboratory of Basic and Clinical Translational of Bone and Joint Diseases in Shaanxi Provincial, Xi'an, Shaanxi, China

**Keywords:** Osteoarthritis, Mechanobiology, Biomaterials, Mechanical microenvironment, Disease modification

## Abstract

Osteoarthritis (OA) is a whole-joint disease characterized by progressive cartilage degeneration, synovial inflammation, and subchondral bone remodeling. Disrupted mechanical homeostasis contributes to these interconnected changes by altering load distribution, tissue deformation, and cellular responses to mechanical stimuli. Inflammation, aging, and matrix remodeling further modify cellular mechanosensitivity, allowing similar mechanical inputs to produce distinct biological outcomes. Conventional biomaterials have largely emphasized defect filling, structural support, and matching the mechanical properties of native cartilage. However, reproducing tissue stiffness does not necessarily restore the dynamic mechanical microenvironment experienced by cells, underscoring the need to regulate pathological mechanical information rather than static material properties alone. This review examines the multiscale mechanical pathology of OA and the mechanosensing and mechanotransduction processes that connect local physical cues with disease progression. It synthesizes biomaterial design strategies involving spatially regulated mechanics, viscoelastic adaptation, mechanical energy conversion, external physical actuation, and mechanically coupled therapeutic delivery. These approaches are discussed in relation to chondrocyte homeostasis, stem cell fate, synovial immune responses, osteochondral remodeling, and aging-associated dysfunction. A phenotype-guided framework is proposed to align material functions with priority mechanical abnormalities across different disease stages and patient-specific pathological contexts, while recognizing that multiple pathological features often coexist and evolve within individual joints. We further identifies requirements for disease-modifying evaluation, including causal mechanistic evidence, physiologically relevant dynamic testing, whole-joint functional outcomes, and durability after material degradation or treatment withdrawal. Together, these perspectives connect mechanobiological principles with rational material design and outline a path toward sustained OA intervention.

## Highlights

• This review reframes osteoarthritis as a multiscale disorder of mechanical homeostasis rather than isolated cartilage degeneration.
• Mechanobiology-instructive biomaterials regulate how mechanical information is transmitted, transformed, and interpreted by joint cells.
• Mechanobiology-instructive biomaterials reshape cell and tissue states across cartilage, synovium, subchondral bone, and the osteochondral unit.
• A phenotype-guided framework links dominant mechanical pathology to material selection and disease-modifying evaluation.

## Graphical abstract

**Figure:**
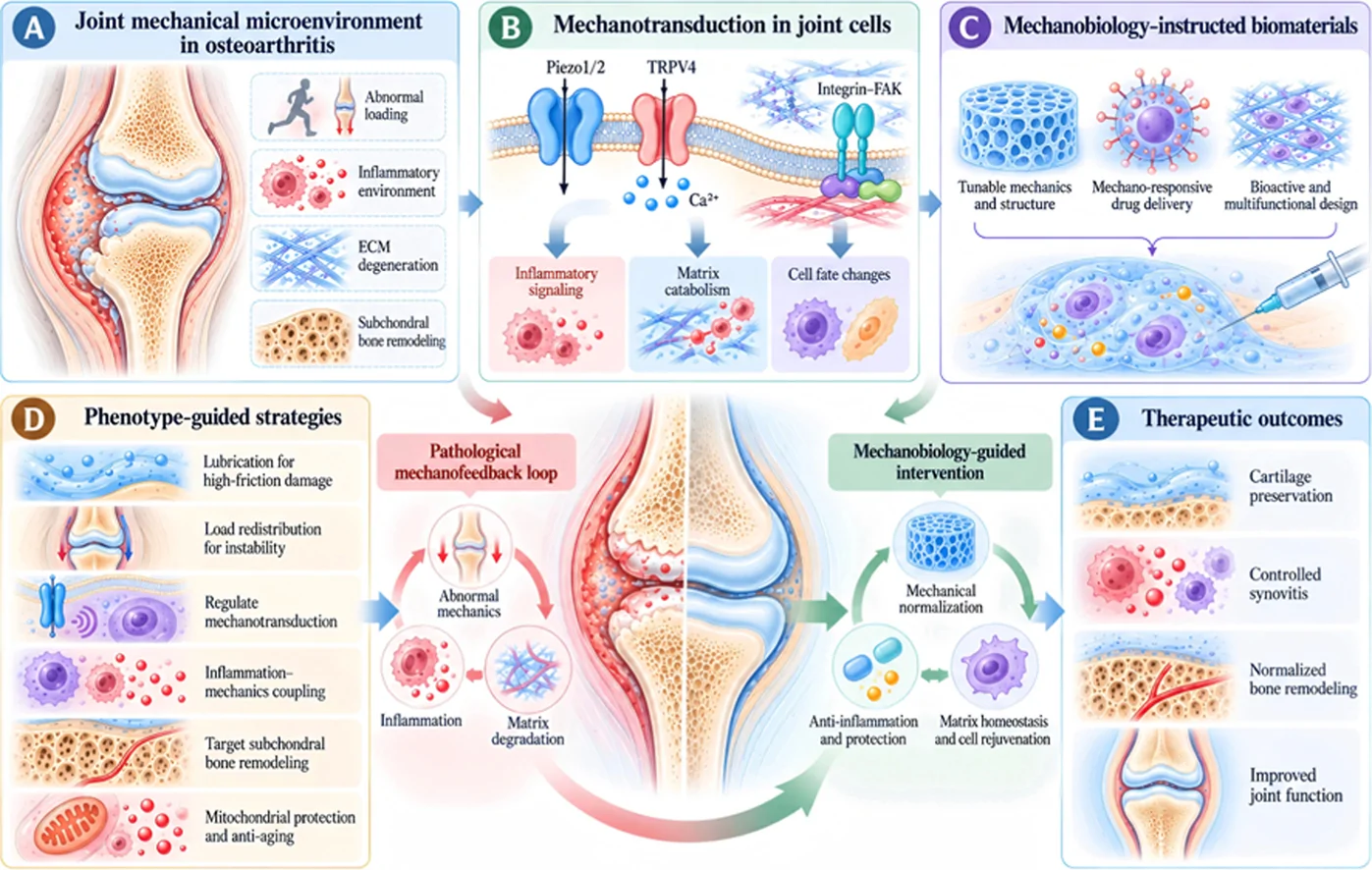

## 1 Introduction

Osteoarthritis (OA) is one of the most common chronic joint diseases and a major cause of pain and movement impairment. Its global disease burden continues to rise with population aging and increasing risk factors such as obesity [1], [2], [3]. Current clinical treatment mainly includes health management, nonsteroidal anti-inflammatory drugs, intra-articular injections, and joint replacement for patients with end-stage disease. These approaches can improve joint function in some patients by reducing inflammation and pain. However, their disease-modifying effects on established tissue degeneration remain limited [4], [5]. As understanding of OA pathology has advanced, OA is no longer regarded as simple articular cartilage wear. It is now considered a condition involving collective changes in cartilage, synovium, subchondral bone, and other joint tissues [6], [7]. These organizations do not change independently, but rather continuously interact with each other on both mechanical and biological levels. Within this pathological network, the abnormal mechanical environment has a particular role. Joints are chronically load-bearing and in motion, and tissue function depends on stable and finely tuned load distribution. Trauma, obesity, joint instability, malalignment, and age-related changes in tissue mechanics can redistribute loads within the joint. This drives local stress, strain, and tissue deformation outside normal ranges. Persistent abnormal loading not only changes the load-bearing state of cartilage. It also affects synovial inflammation, subchondral bone remodeling, and the mechanical boundaries of the entire osteochondral unit [8], [9]. Meanwhile, inflammation, aging, and metabolic abnormalities reduce the ability of joint tissues to maintain homeostasis and adapt to mechanical stimuli. Mechanical inputs that could originally be buffered by tissue and regulated by cells more readily become persistent injury signals [10], [11], [12], [13]. Therefore, an abnormal mechanical environment is not merely a secondary phenomenon after OA tissue degeneration. It is an important pathological factor involved in disease onset and sustained progressio.

The influence of the mechanical environment on OA progression must ultimately be converted into sustained biological effects through cellular sensing of and responses to physical stimuli. Macroscopic loads borne by the joint during motion are not transmitted directly to cells in the same form. They are redistributed by tissue structure, extracellular matrix, and pericellular matrix. Normal articular cartilage has characteristic multiphasic and time-dependent mechanical properties. Interstitial fluid and the solid matrix together bear external loads. Fluid migration and matrix deformation create local mechanical states that change over time [14], [15]. Therefore, what cells actually sense is not the external load borne by the whole joint. It is the deformation, tension, fluid flow, and spatial confinement produced after regulation by local tissue structure. The pericellular matrix lies between chondrocytes and the distant extracellular matrix. Its composition and mechanical properties strongly affect how external loads are transmitted to cells [16], [17]. During OA progression, proteoglycan loss, collagen network destruction, and pericellular remodeling alter this local mechanical filtering capacity. As a result, cells experience mechanical stimuli different from healthy conditions under similar macroscopic loads [18], [19], [20]. Cells can further convert these physical changes into cytoskeletal reorganization, metabolic changes, and altered gene expression. These effects then feed back into matrix synthesis, tissue structure, and local mechanical state [21]. OA mechanical pathology therefore has clear multiscale features. Load changes at the tissue level can propagate down to cellular and subcellular scales. Changes in cell state can further affect the tissue mechanical environment through matrix remodeling and inflammatory responses. More importantly, cellular responses to mechanical stimuli are not fixed. Mechanical stimuli within the physiological range usually help maintain tissue homeostasis. However, when stimulus intensity, duration, or local mechanical context changes, the biological outcome may shift toward catabolism and injury. Inflammation and aging also alter the mechanosensitive state of cells. The same external load can therefore produce different outcomes at different disease stages [22], [23]. Thus, mechanical abnormalities in OA cannot be described only by joint load magnitude or average tissue stiffness. We also need to understand how external loads are converted into local mechanical information through tissue structure, and how disease states change the way cells read this information. Only at this level can mechanical abnormalities be truly linked to cellular state changes and OA progression.

Recently, as understanding of cellular mechanosensing and its relationship with OA progression has deepened, the role of mechanical factors in tissue repair is no longer limited to providing structural support [24], [25]. They are increasingly regarded as important biophysical signals that can actively regulate cell state. Mechanobiology has thus become an important theoretical basis for understanding OA pathology and guiding material design. Its focus has expanded from what loads tissues bear to how cells sense, transmit, and respond to this mechanical information, and how these processes affect tissue homeostasis and disease progression. Traditional cartilage repair materials usually emphasize defect filling, structural support, cell adhesion, and matching of material mechanical properties to native cartilage. These factors remain basic requirements for materials to function in the joint environment. However, obtaining a static modulus similar to native cartilage does not guarantee that cells will experience a near-physiological mechanical microenvironment. Cells can actively sense the mechanical properties of the surrounding matrix. They adjust their morphology, cytoskeletal state, and functional phenotype according to external mechanical resistance [26], [27]. Native extracellular matrix is also clearly viscoelastic. Its mechanical response changes continuously with loading time, fluid migration, and structural rearrangement. The mechanical environment experienced by cells is therefore dynamic [28]. When initial stiffness is similar, changes in matrix stress relaxation alone can significantly affect cell behavior. This indicates that time-dependent mechanical properties can themselves be important biophysical regulators [29]. For cartilage, a tissue that bears cyclic loads over long periods, the mechanical resistance, local deformation, and spatial confinement formed during sustained compression and tissue remodeling also affect cell state and new matrix formation [30]. Therefore, the design focus of mechanobiology-instructed biomaterials should not stop at increasing material strength or replicating a normal tissue mechanical parameter. It should further consider how materials change the mechanical microenvironment actually experienced by cells. Material stiffness, viscoelasticity, stress relaxation, permeability, and spatial structure thus gradually shift from traditional engineering parameters to biophysical information capable of regulating cell state [31], [32], [33]. For OA, a disease in which mechanical homeostasis is already disrupted, material design goals accordingly expand from passive load bearing and structural matching to mechanical environment regulation. By altering the transmission of abnormal mechanical signals, materials can re-establish physical conditions more favorable for tissue homeostasis and repair. In this context, mechanobiology provides new research directions for OA biomaterials. However, material design still needs clearer links to the mechanical pathology of the disease itself. OA does not have a completely uniform mechanical abnormality. Different etiologies and disease stages can produce different tissue loading states and cellular mechanical responses. Trauma-related changes in joint stability, age-related tissue mechanical degeneration, and obesity-related load and metabolic changes may all expose materials to different local mechanical environments [8], [34], [35]. In recent years, OA research has also begun to focus more on pathological phenotypes and molecular endotypes. This aims to explain differences in disease progression and treatment response among patients at the mechanistic level [36]. This understanding suggests that mechanobiology-instructed biomaterials cannot use near-normal tissue mechanical parameters as the sole design endpoint. They need to clarify whether the mechanical features they regulate correspond to a specific pathological state. At the same time, improvement in macroscopic material mechanical properties cannot be directly equated with restoration of the cellular mechanical environment. The mechanical effects produced by materials still need to pass through tissue structure and cellular mechanosensing to generate final biological effects. Research on such biomaterials therefore needs to gradually establish continuous links among material physical properties, local mechanical state, cellular responses, and tissue function.

Based on this understanding, this review explores the multiscale changes in the mechanical environment of normal joints and during the progression of OA from the perspective of OA mechanopathology, and outlines the mechanisms by which joint cells perceive and integrate this mechanical information(Fig. 1). Building on this foundation, the review further analyzes how biomaterials modulate cell function through controlled mechanical properties and discusses different material design strategies from the perspective of mechanical information processing. Subsequently, this overview focuses on the responses of various joint cells and tissues to mechanical cues from materials. Drawing on different mechanical pathophysiological contexts of OA, it establishes a correspondence between material functions and therapeutic goals, and proposes a framework for mechanobiology-instructed biomaterials to achieve disease modification. Through this framework, this review aims to establish a clearer trajectory from mechanical abnormalities in OA to material design, providing a theoretical foundation for advancing OA biomaterials from tissue mechanics simulation toward the regulation of pathological mechanical environments.

**Fig. 1:**
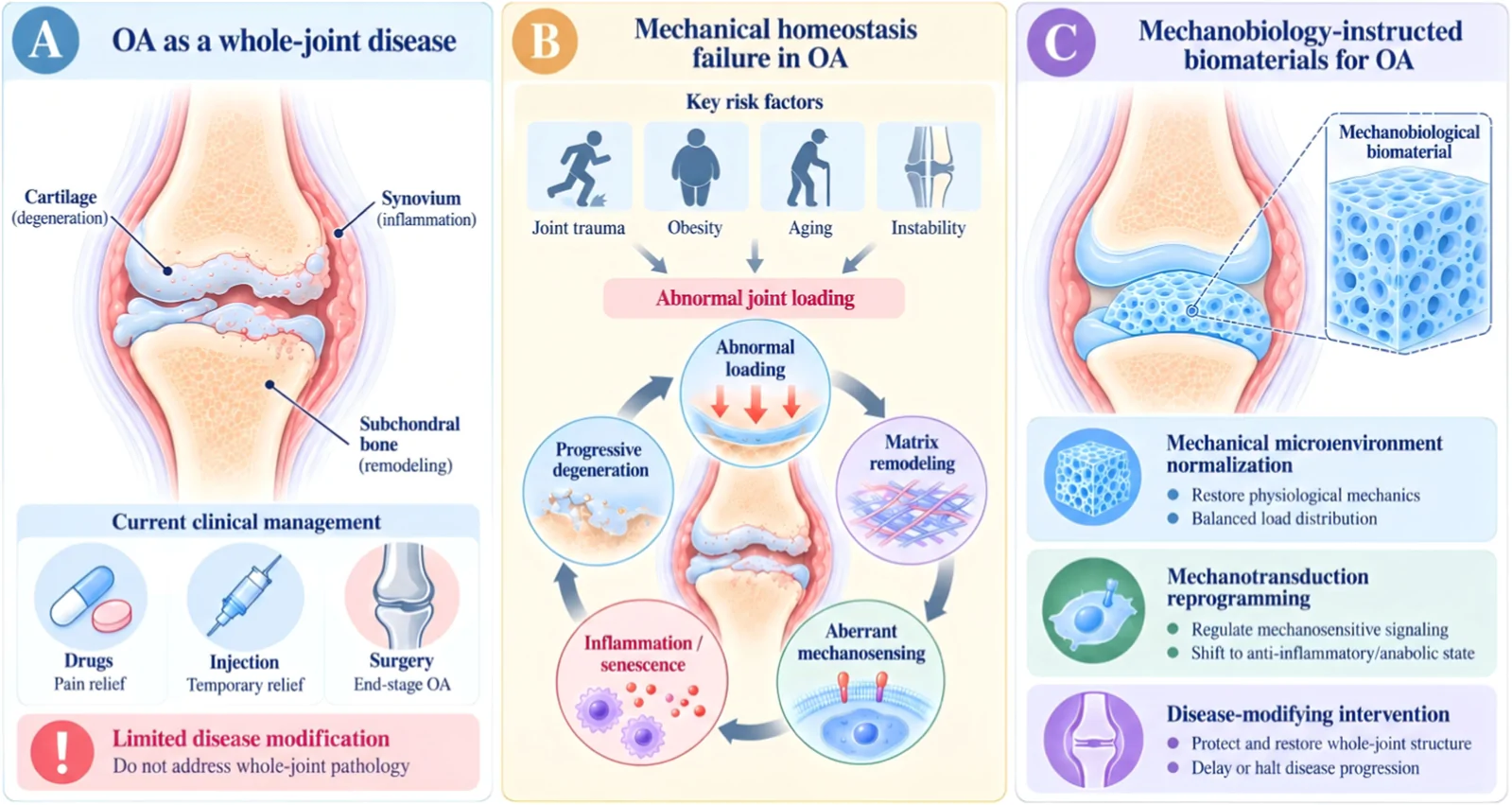
**Mechanobiology-instructed biomaterials for osteoarthritis disease modification.** (A) Whole-joint OA pathology involving cartilage degeneration, synovial inflammation, and subchondral bone remodeling. (B) Disrupted mechanical homeostasis and the resulting feedback among abnormal loading, matrix remodeling, mechanosensing, and inflammation. (C) Mechanobiology-instructed biomaterials targeting pathological mechanical cues and downstream mechanotransduction to support disease modification.

## 2 Multiscale mechanical pathology of osteoarthritis

### 2.1 Multiscale mechanical homeostasis in healthy joints

Articular cartilage is a hydrated multiphasic tissue. Its mechanical behavior arises from coupling between the collagen-proteoglycan solid network and interstitial fluid. Classical biphasic theory shows that after load application, a substantial portion of pressure is initially borne by interstitial fluid. As fluid migrates, load is redistributed to the solid matrix. Cartilage therefore exhibits creep and stress relaxation [37]. For cartilage materials, this means that instantaneous elastic modulus describes only part of the mechanical behavior. Before tissue-level stress reaches chondrocytes, it must be redistributed by the pericellular matrix. This region is rich in type VI collagen and proteoglycans. It has a composition and mechanical features different from the distal extracellular matrix. Alexopoulos et al. measured human chondrocyte pericellular matrix and found that OA significantly alters its biphasic mechanical properties. This suggests that chondrocytes may be exposed to an abnormal mechanical microenvironment before obvious macroscopic tissue destruction [38]. Szafranski et al. further showed that cartilage compression deforms chondrocytes and intracellular organelles. Organelle deformation does not simply correspond linearly to tissue strain [39]. Thus, from whole-knee load to local matrix strain, and then to membrane tension, cytoskeletal deformation, and nuclear deformation, there are multiple levels of mechanical information recoding.

### 2.2 Remodeling of the mechanical microenvironment in OA

Early proteoglycan loss in OA reduces fixed charge density and changes tissue water absorption and permeability [40]. Collagen network destruction weakens tensile restraint. This increases lateral expansion and local cellular strain during compression [41]. With surface fibrillation, fissures, and cartilage thinning, load distribution becomes more regionally uneven. Therefore, OA-related changes in cartilage stiffness are spatially heterogeneous and vary with disease stage and anatomical location, superimposed on the intrinsic depth-dependent mechanical properties of cartilage [42], [43]. This heterogeneity can directly alter how cells read their material environment. Different three-dimensional stiffness environments can change chondrocyte morphology, matrix synthesis, and cellular mechanical state [44]. Extracellular matrix ligands, pore structure, and cell composition further modify this stiffness effect. Thus, even within the same macroscopic modulus range, different material chemistry and local structure can produce completely different cellular mechanical feedback. The osteoarthritic mechanical microenvironment is better understood as a combination of multiple changing parameters, not as a single modulus deviation from normal [45]. At the same time, the time-dependent behavior of cartilage has received increasing attention. Three-dimensional dynamic network studies show that stress relaxation rate and fluid migration can independently change sustained mechanical resistance around cells even when initial stiffness is similar [46]. This further affects matrix deposition and tissue formation. These results indicate that the osteoarthritic mechanical microenvironment should not be described by a single elastic parameter. It should also consider relaxation time, permeability, fluid migration, and local strain history.

### 2.3 Pathological mechanocoupling across the osteochondral unit

Subchondral bone is not a passive support layer beneath cartilage. When cartilage loses buffering capacity, local load transferred to subchondral bone increases. On the other hand, trabecular remodeling and sclerosis in subchondral bone alter the effective stiffness of the osteochondral interface [47]. This further reshapes cartilage stress distribution. Li et al. found that Forkhead box C1 (FOXC1) and Piezo1 are elevated in human osteoarthritic subchondral bone and in cell experiments. They promote pathological osteoblast states through Piezo1 and YAP-related signaling. This suggests that mechanosensitive pathways in subchondral bone directly participate in OA [48]. Further animal and cell studies show that abnormal mechanical loading can promote subchondral bone remodeling through osteoblast Piezo1, intracellular calcium, and JAK2/STAT3signaling. This is associated with uneven tibial plateau depression [49]. Such studies indicate that material design for the osteoarthritic mechanical environment cannot ignore subchondral bone. Simply adding a soft material to the joint surface cannot fully address cross-tissue load abnormalities. Material design has also begun to mimic this cross-tissue mechanical continuity. By gradually changing the inorganic phase, porosity, and network density from the cartilage side to the bone side, local modulus and load-bearing mode can transition continuously. This reduces stiffness mismatch at the interface of traditional bilayer structures [50]. This approach provides a material basis for reconstructing osteochondral load transfer. It also means that the osteochondral interface is no longer only a histological layer. It becomes a mechanical transition region that requires active design [51].

### 2.4 Reciprocal coupling between mechanical stress and inflammation

Mechanobiology in OA is not independent of inflammation. Inflammatory mediators can increase the expression and mechanical sensitivity of mechanosensitive channels. This makes chondrocytes produce stronger calcium signals and injury responses to the same deformation [52]. In turn, sustained mechanical damage promotes the release of inflammatory mediators and matrix degradation products. Therefore, mechanical abnormality and inflammation are not two parallel pathways. They form a coupled process that can amplify each other [53]. Studies in the other direction show that moderate mechanical stimulation can also suppress some inflammatory programs [54], [55]. This is accompanied by dynamic adjustments in primary cilia length, cytoskeleton, and mechanosensing structures. Together, these results indicate that the key problem in OA is not mechanical stimulation itself. It is the dose, duration, cell state, and specific sensing mode of mechanical stimulation. Different load intensities may even produce opposite biological outcomes through the same mechanical network [56]. Materials can directly regulate this process. Interfacial lubrication can reduce sustained shear and cartilage surface damage. Dynamic networks can reduce long-term mechanical restriction. Anti-inflammatory or antioxidant components can further alter the pathological cell environment. Their value is not merely to increase the number of material functions. Physical mechanical protection and inflammation regulation act at different positions in the pathological feedback loop. This makes them complementary.

### 2.5 Mechanical memory and persistence of pathological cell states

The impact of mechanical loading does not stop immediately after load removal. Sustained mechanical overloading can induce oxidative and mitochondrial stress and promote chondrocyte senescence [56], [57]. The senescence-associated secretory phenotype of senescent chondrocytes can then continuously promote inflammation and matrix degradation. This converts short-term mechanical injury into long-term tissue changes. Material studies have begun to address this persistent cell state. For age-related OA, Yan et al. constructed a tissue-adhesive polyphenol-coated nanogene hydrogel. It was used to regulate senescent chondrocyte states and improve cartilage lesions in aged OA model [58]. Although the main action of this material is gene and anti-aging therapy rather than direct mechanical regulation, it suggests that changing the mechanical environment alone may not be enough to reverse an established senescent phenotype. Mechanical memory has also entered cartilage biomaterial research. Studies using microgrooved hydrogels to create specific cellular mechanical states show that cells can form persistent phenotypes in a preset physical environment. This links mechanical preconditioning to cartilage repair [59]. Such research is still early. It is not yet sufficient to treat mechanical memory as an independent OA therapeutic strategy. However, it may explain why long-term mechanical abnormalities can continue to affect cells even after structural load is improved.

### 2.6 Self-reinforcing mechanical pathology and therapeutic entry points

Taken together, existing original studies suggest that osteoarthritic mechanical pathology can be understood as persistent mutual reinforcement among tissue-level load changes, pericellular matrix remodeling, abnormal mechanical perception, and inflammation-metabolism imbalance(Fig. 2). Biomaterials can intervene at different levels. These include reducing joint surface friction, restoring local viscoelastic environment, changing cell traction resistance, regulating mechanosensitive ion channels, using mechanoelectrical conversion to create new biophysical stimulation, or controlling drug release under abnormal mechanical conditions. This pathological structure also means that no single mechanical parameter applies to all OA types. Post-traumatic joint instability and age-related OA differ clearly in initial mechanical conditions and cell states. Therefore, mechanobiology-instructed material design needs to shift gradually from copying normal cartilage parameters to correcting specific pathological mechanical phenotypes.

**Fig. 2:**
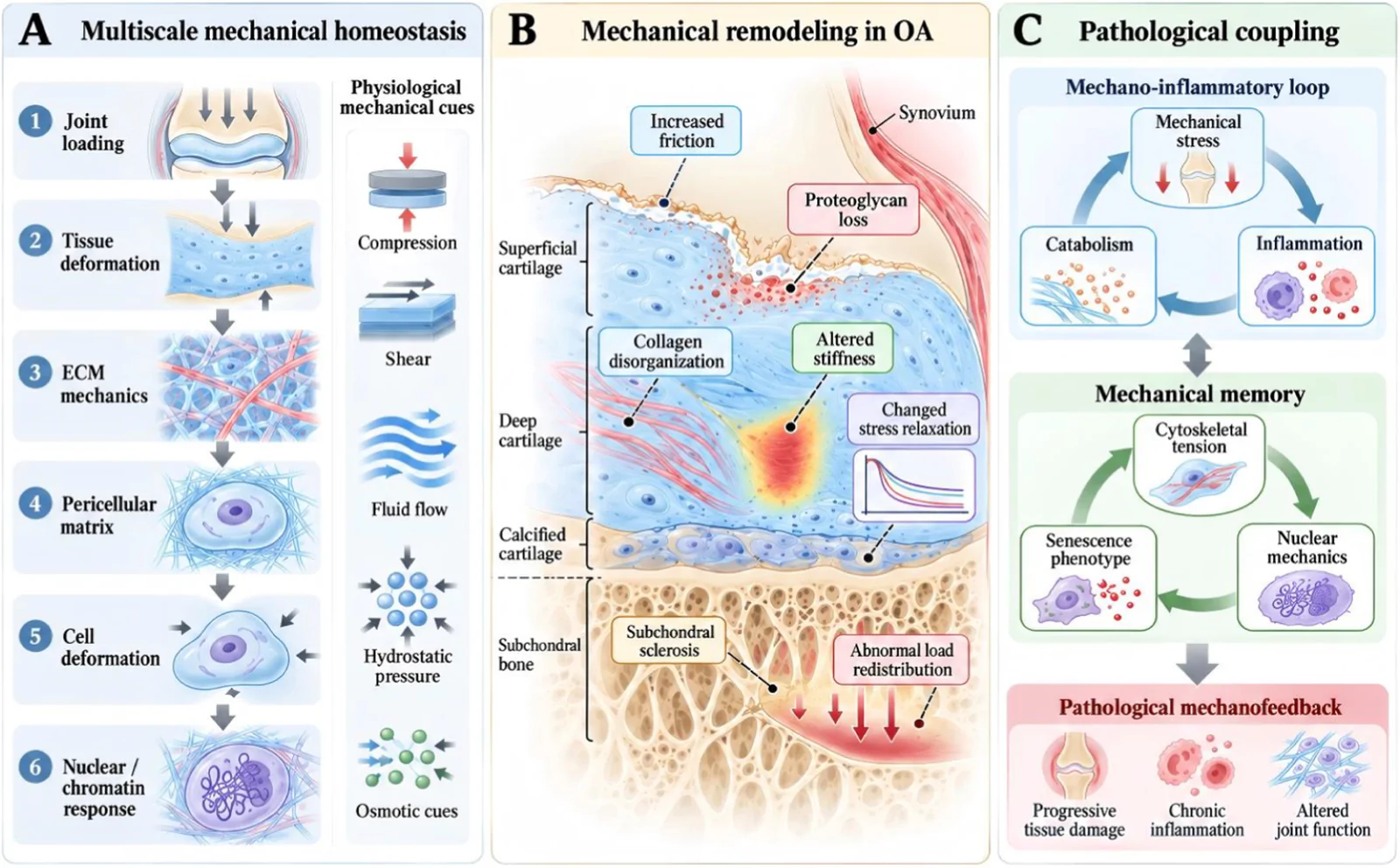
**Multiscale mechanical pathology and pathological mechanocoupling in osteoarthritis.** (A) Physiological load transmission across tissue, extracellular matrix, pericellular matrix, cellular, and nuclear levels. (B) OA-associated changes in friction, matrix mechanics, stress relaxation, subchondral bone, and load distribution. (C) Mechano-inflammatory feedback and mechanical memory contributing to persistent cellular dysfunction and whole-joint degeneration.

## 3 Cellular mechanosensing and mechanotransduction in OA

Articular cells are not passive recipients of tissue load. They continuously interpret the surrounding physical environment through membrane mechanosensitive ion channels, integrin adhesions, the cytoskeleton, primary cilia, and nuclear mechanical structures [60]. Tissue-level compression, shear, and fluid movement are redistributed by the extracellular matrix and pericellular matrix before reaching cells. Therefore, what cells actually read is local membrane tension, fluid shear, adhesion resistance, and cell and nuclear deformation [61]. Different mechanosensing systems further crosstalk. Mechanical stimuli are ultimately integrated into calcium signals, cytoskeletal tension, kinase activity, and transcriptional states. Material stiffness, viscoelasticity, permeability, and dynamic deformation are also converted into cellular effects through this continuous network.

### 3.1 Mechanosensitive ion channels: rapid decoding of mechanical inputs

Mechanosensitive ion channels on the chondrocyte membrane can convert membrane tension, osmotic changes, and local deformation into ion currents within a short time. They therefore form a direct interface for mechanical information entering cells. Dynamic compression can regulate chondrocyte metabolism through TRPV4. When this channel is blocked, mechanical loading-induced anabolic responses and extracellular matrix formation are markedly reduced. Channel activation can partially replicate the cellular effects of dynamic mechanical stimulation [62]. This indicates that chondrocytes do not respond to external compression through nonspecific deformation. They use specific mechanosensitive channels to selectively encode dynamic load. Piezo1 shows more obvious pathological sensitivity under higher-amplitude mechanical stimulation and inflammatory conditions [63]. Inflammatory mediators can increase Piezo1 expression and enhance its mechanosensitivity. This makes chondrocytes produce stronger calcium signals and cell injury under the same mechanical deformation [52]. This inflammation-induced mechanical sensitization makes Piezo1 an important connection point for mutual amplification between mechanical injury and inflammation. It also shows that the mechanical range tolerated by cells changes in disease states. Piezo channels and TRPV4 cannot be simply divided into injurious and protective categories. Calcium signals produced by different channels differ in amplitude, duration, and intracellular spatial distribution. The frequency and duration of mechanical stimulation further alter these signals. Short, cyclic mechanical stimulation and sustained high strain produce different calcium dynamics. Downstream cytoskeletal, mitochondrial, and transcriptional responses may therefore diverge markedly [64]. This mechanical threshold is also regulated by the pericellular matrix. The pericellular matrix can limit excessive chondrocyte deformation. After it gradually forms, cell deformation caused by the same macroscopic compression decreases. When its glycosaminoglycan components are degraded, cells again show greater deformation under the same load [65]. Further loading experiments show that chondrocyte death is related to both strain amplitude and duration. Pericellular matrix formation can increase the range of compression tolerated by cells. Therefore, mechanosensitive ion channels do not recognize external joint load itself. They recognize the cell membrane mechanical state after local matrix filtering. From a biomaterial design perspective, this means that describing how much deformation a material can produce is not sufficient. What determines cellular effects may be the membrane tension and calcium signals formed after deformation passes through the material and pericellular matrix. It also depends on whether these signals remain within a physiological response range.

### 3.2 Integrin–FAK signaling: matrix mechanics to cytoskeletal tension

Unlike mechanosensitive ion channels, which mainly recognize membrane tension, the integrin system recognizes external mechanical resistance through physical connections between cells and matrix. After integrins bind extracellular matrix, they connect to the actin cytoskeleton through focal adhesion proteins such as talin and vinculin. Through FAK and Src family kinases, they convert external pulling forces into intracellular phosphorylation signals [60]. Therefore, cells sense not only how hard a material is. They also sense whether adhesion sites are stable and how much reaction force the material provides when cells generate traction [66]. Experiments with chondrocytes in matrices of different stiffness further show that matrix mechanics can change the cell's own mechanical properties. As surrounding matrix stiffness increases, chondrocyte spreading area and irregular morphology increase. Actin structures become more prominent. Their elastic modulus, relaxation modulus, and apparent viscosity also change [67]. This indicates that the material mechanical environment not only changes external forces on cells. It also enables cells to actively adjust their own mechanical state, creating new mechanical feedback. The cell's own elasticity, viscosity, and cytoskeletal organization can therefore be viewed as a secondary mechanical phenotype shaped by long-term external matrix mechanics. Integrin-related mechanical signaling has clear load dependence. Moderate mechanical input can promote adhesion and cytoskeletal responses that maintain cartilage homeostasis. Sustained or excessive mechanical stimulation may form a high-tension state through excessive focal adhesion maturation and cytoskeletal contraction. Thus, changes in FAK activity alone cannot be simply interpreted as protective or injurious. Their biological effects need to be analyzed together with load dose and initial cell state [68]. This process is also spatially regulated by the pericellular matrix. Finite element analysis shows that the modulus difference between the pericellular matrix and distal extracellular matrix can significantly alter stress and strain inside chondrocytes. Its lower permeability can reduce fluid flux near cells. OA-related pericellular matrix softening and increased permeability markedly alter this local microenvironment [69]. Multiscale models further show that even when macroscopic cartilage structure has not changed noticeably, local softening of the pericellular matrix and adjacent extracellular matrix alone can significantly increase chondrocyte shear strain [70]. Therefore, integrins and focal adhesions read local mechanical resistance after microscale matrix remodeling. They do not read the average stiffness of joint tissue. This also explains the potential advantage of dynamic materials. Fixed covalent networks maintain strong reaction forces after sustained cell traction. Rearrangeable networks can gradually release local stress. The effective mechanical resistance experienced by integrins and focal adhesions decreases over time. Material viscoelasticity can therefore continuously change cell adhesion mechanical states without large changes in initial modulus.

### 3.3 RhoA–ROCK–YAP signaling: cytoskeletal tension to transcriptional control

Mechanical signals generated by focal adhesions further regulate myosin contraction and actin stress fibers through RhoA and ROCK. This converts external matrix resistance into intracellular tension [71]. Chondrocytes themselves are viscoelastic. Their mechanical behavior depends on actin, microtubules, and intermediate filaments [72]. Therefore, the cytoskeleton not only provides structural function. It is also a major channel for mechanical information transmission. Changes in matrix stiffness can significantly alter this tension state. Stiffer matrices promote cell spreading and stress fiber formation. This increases cell elasticity and viscosity. Softer environments better maintain a round chondrocyte morphology. This cellular mechanical adaptation caused by external matrix stiffness provides a physical basis for subsequent changes in mechanosensitive transcription factors. YAP is one of the more deeply studied nodes connecting cytoskeletal tension to nuclear transcriptional states. Chondrocytes on matrices of different elasticity show distinctly different YAP localization. Stiffer matrices more easily promote its nuclear localization and are accompanied by weakened cartilage phenotype. Reducing its activity helps maintain cartilage-related gene expression [73]. Further simulation of different pathological stiffness shows that matrix stiffening can continuously enhance chondrocyte YAP activity and is associated with loss of cell phenotype. Modulating this mechanotransduction node in animal models can reduce cartilage degeneration [74]. This indicates that matrix mechanical changes are not only accompanied by disease progression. Matrix mechanical changes can directly participate in shaping cell states through cytoskeleton-nuclear mechanical signaling. Notably, YAP action is highly context-dependent. Its activity is regulated by cell type, cell density, cytoskeletal tension, and differentiation stage [75]. Completely shutting down mechanosensitive transcription may also affect cell migration and normal tissue renewal. Therefore, for mechanobiology-instructed materials, a more reasonable goal is to control cytoskeletal tension and nuclear mechanical signaling within an appropriate range. It is not to define a mechanical pathway as a pathological switch that needs continuous suppression. This mechanism also shows that the effect of material stiffness on cells is not a single step from stiffness to YAP. It is transmitted stepwise through integrin adhesion, RhoA/ROCK, actin tension, and nuclear mechanical state. Multiple intermediate steps between material parameters and final transcriptional outcomes can be dynamically readjusted by mechanics.

### 3.4 Primary cilia: integration of compression and fluid-flow signals

Primary cilia extend from the chondrocyte surface into the pericellular space. They directly contact tissue fluid and pericellular matrix. Therefore, they can sense compression, fluid movement, and local spatial constraints [76]. Unlike the rapid opening of membrane ion channels, the length and structure of primary cilia can themselves remodel according to the mechanical environment [77]. This gives them both mechanosensing and mechanical adaptation features. Three-dimensional agarose models show that cyclic compression can significantly reduce the incidence and length of chondrocyte primary cilia. After load removal, some changes recover [78]. This indicates that primary cilia are not fixed sensory structures. Their state can record recent mechanical stimulation received by cells. The link between mechanical loading and inflammation also involves primary cilia. Regulation of cartilage inflammatory signals by mechanical stimulation is related to HDAC6, intraflagellar transport, and changes in cilia length. This suggests that primary cilia participate in crosstalk between mechanical and inflammatory signals [55]. The mechanical environment experienced by primary cilia is highly dependent on the pericellular matrix. Normal pericellular matrix modulus lies between that of chondrocytes and distal extracellular matrix. It can significantly shield and redistribute tissue strain. After cartilage degeneration, this region's elastic modulus decreases and permeability increases. This alters strain and fluid environment near cells [19]. Early cell culture experiments also show that as chondrocytes gradually form their own pericellular matrix, cell deformation caused by the same macroscopic compression gradually decreases. After enzymatic degradation of glycosaminoglycans, this protective effect is markedly weakened [65]. Therefore, the fluid and deformation actually sensed by primary cilia cannot be directly inferred from the overall compression rate of a material. It changes continuously with local matrix maturation, degradation, and fluid migration. For three-dimensional hydrogels and scaffolds, this means that pore structure, permeability, and local fluid migration are as important as stiffness. Even if a material reaches a suitable compressive modulus, if loading produces almost no physiological fluid movement, its mechanical microenvironment may still differ noticeably from native cartilage [79], [80]. Primary cilia therefore provide an important interface linking material fluid mechanics and cellular mechanotransduction [81].

### 3.5 Nuclear mechanotransduction and mechanical memory

Mechanical signals sensed by the membrane and focal adhesions can ultimately be transmitted along the cytoskeleton to the nucleus [82]. The linker of nucleoskeleton and cytoskeleton complex transmits tension generated by actin and other cytoskeletal components to the nuclear envelope and lamina. This allows mechanical stimuli to further affect nuclear shape, chromatin organization, and transcriptional regulation. Nuclear mechanotransduction thus provides a mechanism by which short-term mechanical stimuli can be converted into longer-lasting cell state changes [83]. Chondrocytes and their pericellular matrix have distinctly different mechanical properties. In situ studies show that chondrocytes behave as viscoelastic structures. The pericellular matrix has a mechanical stiffness higher than the cell but noticeably lower than the distal extracellular matrix. This forms a mechanical transition layer from tissue to cell. Compression experiments on single chondrocytes and intact chondrocyte units further show that the pericellular matrix can markedly alter overall deformation and stress relaxation behavior [84]. Therefore, nuclear mechanical state cannot be simply predicted from macroscopic tissue strain. Tissue load is first redistributed by the extracellular matrix and pericellular matrix [85]. It is then transmitted to the nuclear envelope through the cell membrane, focal adhesions, and cytoskeleton. In OA, pericellular matrix softening and increased permeability can elevate local cell compression and fluid flux. This places the nucleus in a different mechanical environment [86]. These changes together indicate that the pericellular matrix is both a buffer layer for load transfer from tissue to cell and an important microscale boundary determining nuclear mechanical state. Material time dependence can further alter this long-term nuclear mechanical input. Fast stress-relaxing networks gradually reduce sustained tension after cell traction. Relatively stable networks keep the cytoskeleton at higher long-term prestress. Thus, even when two materials have similar initial stiffness, the long-term mechanical state experienced by the nucleus may differ. This sustained mechanical tension may affect YAP and chromatin accessibility. It may further participate in cell differentiation, inflammation, and aging programs. Compared with transient mechanosensitive channels, nuclear mechanotransduction better explains why chondrocytes in an abnormal matrix mechanical environment for a long time may maintain their original pathological phenotype after external load is briefly changed. Therefore, regulation of nuclear state by mechanobiology-instructed biomaterials cannot rely only on reducing instantaneous deformation. It must also consider whether the material can reduce abnormal cytoskeletal prestress over the long term and allow cells to gradually establish a stable transcriptional state in a new mechanical environment.

### 3.6 Network integration of mechanotransduction

Chondrocyte mechanosensing is not a linear process from a single receptor to a single transcription factor(Fig. 3). Calcium signals generated by Piezo1 and TRPV4 can alter cytoskeletal and metabolic states. Integrins and FAK control actin tension. ROCK further regulates nuclear mechanical state. The pericellular matrix and primary cilia determine the local amplitude and fluid environment before mechanical signals enter cells. Different levels regulate each other. Therefore, the same tissue-level load can produce different biological outcomes depending on cell state. The pericellular matrix provides a typical example of this network integration. Finite element models show that normal pericellular matrix can shield stress to some extent and limit fluid flux near cells. After its degeneration, cell compressive strain and local fluid flow increase markedly [69]. Thus, a structural change outside the cell can simultaneously affect membrane channels, primary cilia, integrin adhesion, and nuclear deformation. It does not correspond to only one mechanical pathway. Multiscale models further show that before macroscopic tissue properties change noticeably, softening of only the microscale pericellular matrix and adjacent matrix is sufficient to markedly increase chondrocyte shear strain. This means that mechanical pathology can first appear at the cell microenvironment level. Traditional bulk material mechanical testing may not capture this early change. Therefore, mechanistic studies of biomaterials should avoid drawing complete mechanotransduction conclusions based only on an increase or decrease in one mechanorelated protein. A more convincing research path is to keep material chemical composition consistent, change only the target mechanical variable, and simultaneously measure local cell deformation, mechanosensing nodes, and downstream phenotypes. Further combining pathway inhibition, gene knockdown, or rescue experiments can determine whether that mechanical node truly participates in the material effect. Mechanobiology-instructed biomaterials ultimately need to establish a continuous relationship from material physical parameters to local cellular mechanical state, mechanosensing network, transcriptional and metabolic state, and tissue function.

**Fig. 3:**
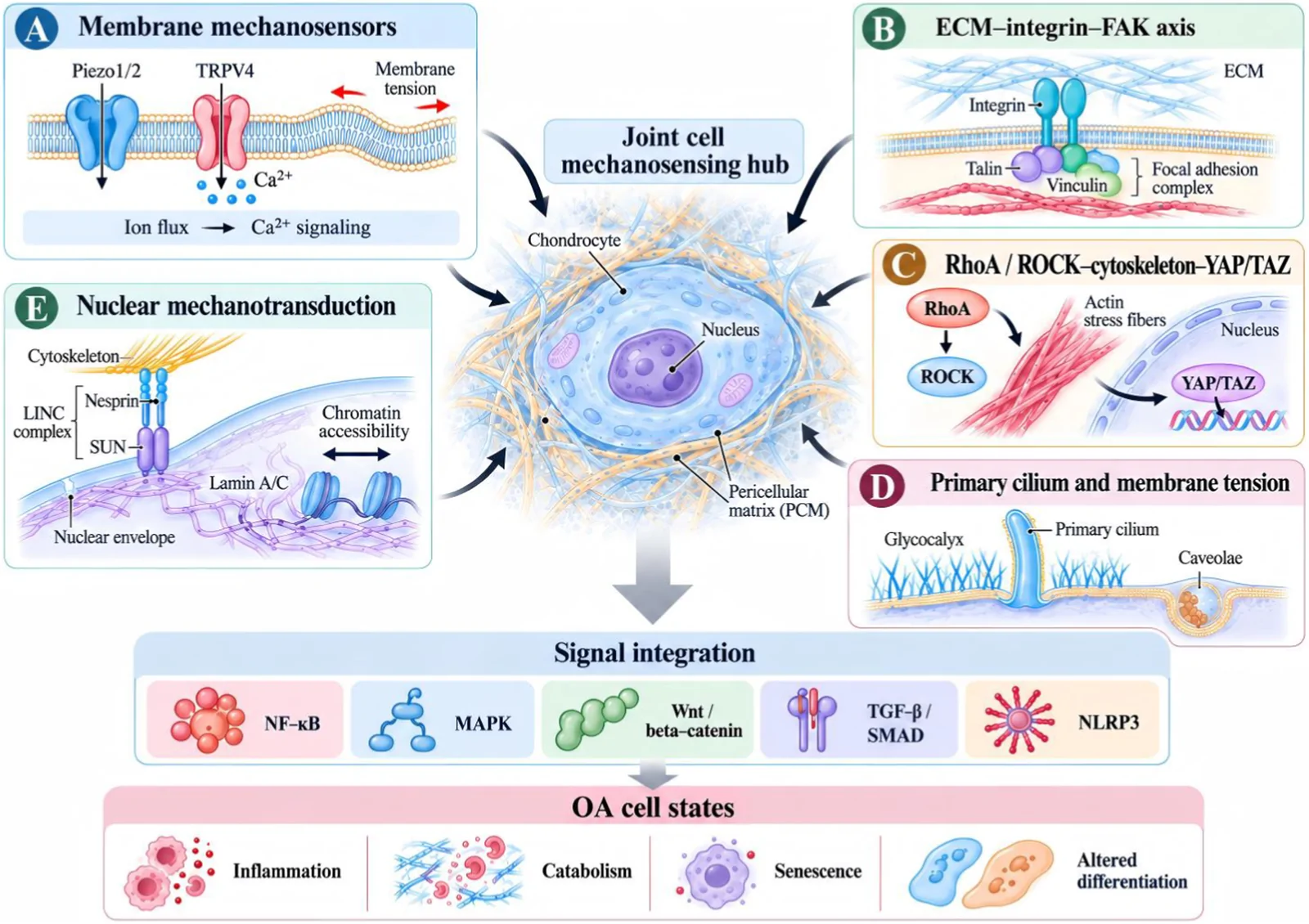
**Integrated mechanosensing and mechanotransduction networks in osteoarthritis.** Mechanical cues are detected by mechanosensitive ion channels, integrin–FAK signaling, primary cilia, and cytoskeletal structures, and integrated through Ca^2+^, RhoA/ROCK, YAP/TAZ, and nuclear signaling pathways. These responses regulate cartilage homeostasis and OA-associated catabolic, inflammatory, and senescent states.

## 4 Mechanobiology-instructed biomaterials: from mechanical encoding to dynamic therapeutic control

The core of mechanobiology-instructed biomaterials is not simply to replicate the macroscopic mechanical parameters of native cartilage. It is to convert material physical properties into mechanical information that can be designed, combined, and regulated. For a joint that continuously experiences compression, shear, fluid migration, and repeated loading, truly meaningful material parameters include baseline stiffness and spatial structure, viscoelasticity and stress relaxation, dynamic bond exchange, mechanoelectrical or ionoelectrical conversion, external physical driving, and mechanically involved therapeutic delivery. Different material forms can share the same mechanical function. The same hydrogel can also simultaneously perform multiple mechanical tasks. Therefore, classifying by functional dimension rather than material name helps establish a unified design language.

### 4.1 Mechanical encoding through stiffness, structure, and spatial gradients

Stiffness is one of the most studied mechanical design variables. Enzyme-crosslinked hydrogels constructed from gelatin hydroxyphenylpropionic acid can achieve different storage moduli by changing network density. This leads to differential chondrocyte proliferation and matrix formation under different mechanical backgrounds [87]. The important materials science point of such systems is that by changing crosslinking degree, comparable mechanical ranges can be established while chemical composition remains relatively stable. This transforms stiffness from a passive measured parameter into an actively set design parameter. However, static mechanical encoding of materials is not equivalent to finding an optimal modulus for all cells and tissue regions. Hydrogels with independently tunable mechanical properties and extracellular matrix components show that biochemical components such as hyaluronic acid and chondroitin sulfate alter how cells read the same stiffness background [88]. Co-culture of adipose-derived stromal cells and chondrocytes further shows noticeable interaction between material mechanical properties and matrix composition [89]. Thus, stiffness is better understood as one dimension in a material design coordinate system, not a single variable that independently determines cell outcomes. Beyond bulk modulus, porosity, fiber orientation, anisotropy, and regional structure also belong to static mechanical encoding. Native cartilage shows noticeable differences from superficial to deep zones in collagen orientation, water content, cell morphology, and load-bearing mode. The osteochondral interface further forms a continuous transition from hydrated soft tissue to mineralized tissue. Multilayer hydrogels can establish differential mechanical environments at different depths by changing polyethylene glycol, hyaluronic acid, chondroitin sulfate, and matrix metalloproteinase-sensitive components [90]. Hierarchical gradient scaffolds further spatially change polymer and ceramic composition. This creates a more continuous structural and mechanical transition between cartilage and bone sides [91]. In recent years, spatial encoding has gradually developed from discrete layering to continuous gradients [51]. Continuous mechanical gradient hydrogels use magnetic fields to regulate the spatial distribution of magnesium-containing hydroxyapatite and iron oxide composite particles. They also establish an inverse gradient of manganese ions. This makes biomaterial mechanics and local biochemical environment change continuously along depth [92]. The key to this design is not simply increasing the number of layers. It is reducing abrupt interfaces between functional regions so that mechanical information changes continuously in space [93]. Directional microchannels and mechanical gradients can be further coupled. This allows materials to simultaneously control load bearing, mass transport, and cell spatial distribution. Beyond cartilage, studies involving hierarchically structured tracheal repair patches and spinal cord scaffolds further illustrate how coordinated material architecture, mechanical properties, and local biological microenvironments can facilitate tissue integration and regeneration [94], [95]. Chiral hydroxyapatite gradient scaffolds show that spatial structural differences can be linked to cell adhesion and mechanosensitive transcriptional states [96]. For biomaterial systems, these studies together promote a shift from structural biomimicry to spatial mechanical information encoding. The core of static mechanical design should therefore not report only final compressive modulus. It should also describe the spatial distribution of mechanical parameters, gradient slope, interface continuity, and whether these parameters persist after long-term culture or degradation.

### 4.2 Temporal adaptation through viscoelasticity, stress relaxation, and dynamic networks

Native cartilage is not an ideal elastic body. After load application, interstitial fluid migration, proteoglycan compression, and collagen network rearrangement cause local stress to decrease over time. Therefore, if a material replicates only instantaneous modulus while ignoring time-dependent behavior, it still cannot reconstruct the mechanical environment actually experienced by cells. Viscoelasticity and stress relaxation extend material design from spatial encoding to temporal encoding. Classical three-dimensional stress relaxation studies tuned alginate ionic crosslinking networks to obtain different relaxation rates at similar initial modulus. This demonstrated that the material time scale itself can independently regulate cell spreading, proliferation, and differentiation [29]. Its biomaterials science contribution is showing that the same initial stiffness can correspond to completely different long-term mechanical resistance. A single-point modulus is therefore insufficient to describe the true matrix experienced by cells. Cartilage-related systems further developed this design principle. Double-network hydrogels composed of gellan gum and polyethylene glycol diacrylate maintain overall mechanical strength while introducing noticeable stress relaxation [97]. Anisotropic gelatin-based materials incorporate both directional structure and time-dependent mechanics. This gives materials tunable mechanical behavior in both spatial direction and time scale [98]. Hyaluronic acid-based biphasic hydrogels further give different regions different relaxation characteristics. This suggests that osteochondral materials can construct not only spatial stiffness gradients but also spatial viscoelasticity and relaxation times [99]. Dynamic covalent networks provide a more direct molecular basis for temporal adaptation. Hydrazone covalent adaptable networks achieve stress relaxation at different time scales through bond exchange kinetics. They allow materials to continuously rearrange under cell traction, new matrix deposition, and external loading [100]. Compared with permanently crosslinked networks, the design focus of such materials is not to keep structure completely unchanged. It is to allow local networks to gradually release constraints while maintaining macroscopic integrity. Cyclic loading further shows that materials themselves act as mechanical filters [101]. When chondrocytes are embedded in different viscoelastic networks and receive the same cyclic compression, they can experience different local mechanical time courses [102]. Therefore, temporal design of joint materials includes at least two scales. One is the instantaneous response of the material to a single load. The other is whether the network undergoes irreversible relaxation, fatigue, or plastic drift after hundreds to tens of thousands of cycles [103]. Thus, faster stress relaxation is not always better. Too-fast network rearrangement may cause the material to lose load bearing and shape retention too early. Too-slow relaxation may create sustained mechanical restriction. A more reasonable design goal is to coordinate relaxation time, permeation behavior, dynamic bond exchange, and material degradation with joint loading cycles and tissue remodeling rate. This allows the material to bear load while gradually adapting to changes in cells and new matrix.

### 4.3 Endogenous mechanical energy harvesting and conversion

Native cartilage undergoes fluid redistribution, ion migration, and streaming potential generation during mechanical deformation, reflecting its intrinsic coupling between mechanical and electrochemical states [104], [105]. Therefore, biomaterials can be designed not only to withstand joint loading but also to harvest endogenous mechanical energy and convert it into electrical or ionic signals. In such systems, joint motion and local deformation serve as mechanical inputs, while the material functions as a transducer that generates secondary physical cues readable by membrane ion channels and other mechanosensitive pathways. Piezoelectric hydrogels provide one strategy for achieving this conversion. Injectable biodegradable hydrogels containing short poly-L-lactic acid piezoelectric nanofibers within a collagen matrix demonstrate that polarization capacity can be integrated into a soft and hydrated network, reducing the mechanical mismatch associated with conventional piezoelectric ceramics [106]. Although this system was activated using ultrasound, it established the feasibility of converting deformation into localized electrical stimulation within a cartilage-compatible matrix. More directly related to endogenous loading, piezoelectric-conductive osteochondral scaffolds can generate potential differences when compressed during joint movement, while conductive components facilitate local signal transmission [107]. This design indicates that piezoelectric output alone does not determine the stimulation received by cells. Conductivity, hydration state, internal resistance, and signal propagation distance are also important design variables. Mechanoionic materials provide another route for endogenous energy conversion that more closely resembles the ionic environment of native cartilage. Mechano-iontronic hydrogels use deformation-induced ion redistribution rather than conventional crystal polarization to generate bioelectric-like signals. Such mechanically generated iontronic electricity can activate Piezo1 and regulate cellular metabolism and chondrogenic responses [108]. More recently, piezo-mimetic ionic hydrogels have been designed to directly convert joint-motion-induced deformation into localized ionic currents through asymmetric ion migration(Fig. 4) [109]. This approach is particularly relevant to mechanically active joints because physiological movement itself provides the energy source without requiring a continuously applied external field. It also shifts material design from maximizing electrical output toward reproducing the coupled mechanical–ionic environment experienced by cartilage cells.Therefore, endogenous energy-converting materials should not be evaluated only by peak voltage or current. More relevant parameters include mechanical activation threshold, conversion efficiency, output amplitude and frequency, ionic conductivity, wet-state signal stability, and output retention during repeated loading. These parameters should be measured under physiologically relevant hydrated and cyclic-loading conditions and linked to downstream mechanosensitive cellular responses. An ideal system should generate reproducible signals within the range of normal joint activity while avoiding uncontrolled output under excessive loading. The design goal is therefore not simply to maximize electrical performance, but to establish a stable input–conversion–response relationship between joint motion, material deformation, local bioelectrical signaling, and cellular adaptation.

**Fig. 4:**
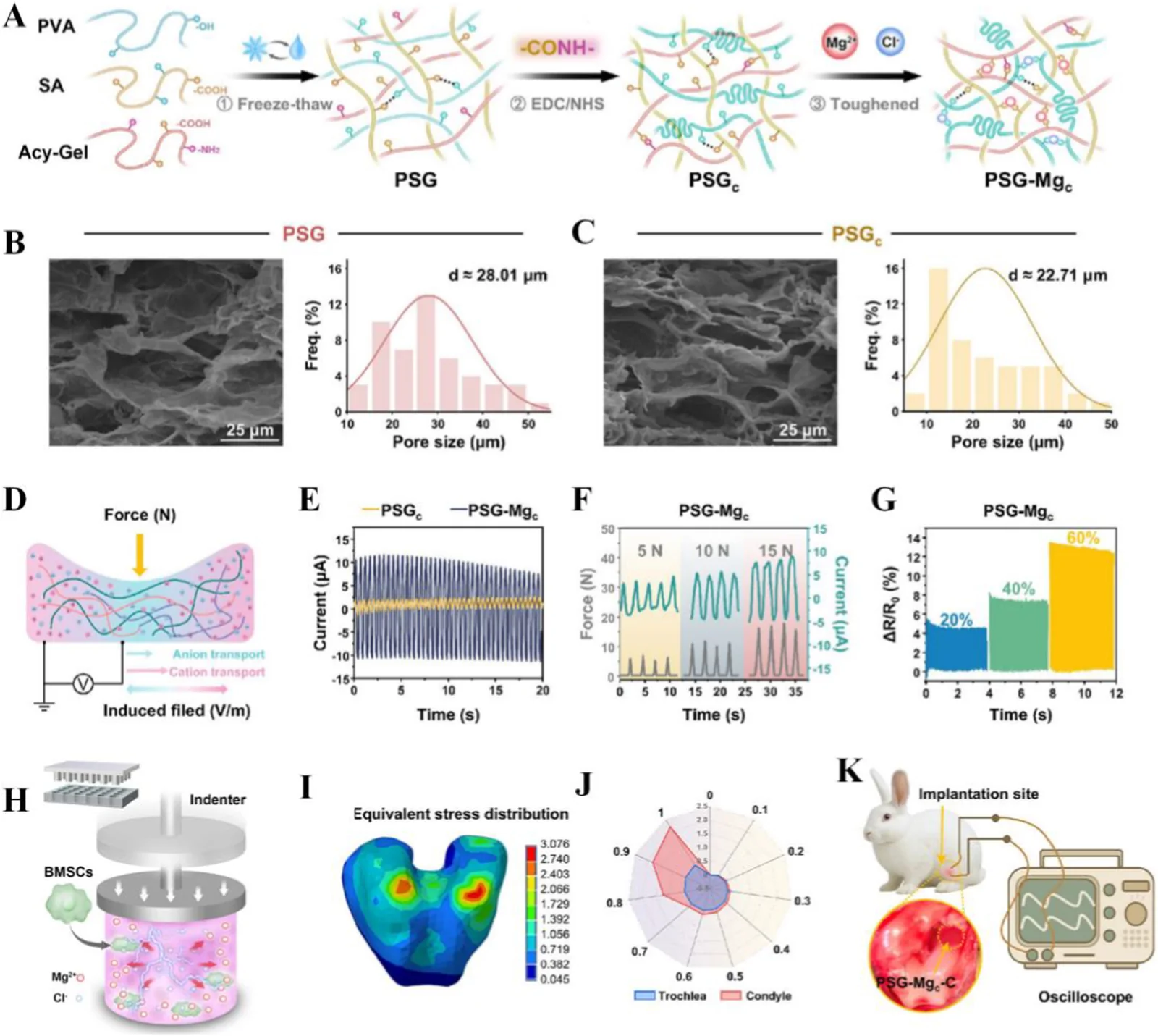
**Mechanically induced electromechanical signaling in piezo-mimetic ionic hydrogels** (A) Schematic illustration of the crosslinked network of the piezo-mimetic ionic hydrogel. (B, C) Cross-sectional SEM images of the hydrogel and the corresponding pore-size distribution. (D) Schematic representation of mechanoelectrical conversion within the hydrogel. (E) Output current responses under mechanical loading. (F) Synchronized current and stress signals. (G) Cyclic strain-sensing performance of the hydrogel. (H) Schematic illustration of the in vitro mechanical stimulation system applied to the piezo-mimetic ionic hydrogel. (I, J) Finite element analysis (FEA)-simulated stress distribution contours in the trochlear and femoral condylar regions. (K) Schematic illustration of the force-dependent electrical signal generated by the piezo-mimetic ionic hydrogel after implantation into an in situ femoral condyle cartilage defect. Reproduced with permission from Ref. [109]. Copyright 2026, Wiley.

### 4.4 Externally actuated mechanical instruction

Self-driven materials rely on patient activity to obtain mechanical energy. They have the advantage of not requiring continuous external devices. However, stimulation dose varies with walking, rest, and exercise intensity. External inputs such as ultrasound, magnetic fields, and pulsed electromagnetic fields allow material mechanical states to be actively adjusted within a set time window. This provides more precise dose control. Ultrasound is essentially a mechanical wave. It can generate cyclic pressure, fluid movement, and microscale deformation within tissue. It therefore has natural coupling with piezoelectric phases [110]. Ultrasound-responsive piezoelectric microspheres integrate barium titanate nanoparticles into gelatin methacryloyl and hyaluronic acid methacryloyl networks. They generate local piezoelectric output under an external acoustic field [111]. Compared with passive systems dependent on joint activity, external ultrasound can independently set stimulation frequency, intensity, and treatment cycle(Fig. 5A) [112]. This makes material mechanical output closer to a prescribable physical dose. External ultrasound itself also causes membrane tension changes, fluid shear, and acoustic radiation force. Therefore, ultrasound-driven materials need to distinguish material-mediated secondary effects from direct acoustic field effects. This issue means that experimental design cannot only include material versus no material controls. It should simultaneously measure acoustic input, material deformation, electrical output, and cellular responses to establish a complete input-output relationship. Magnetic responsive systems use the orientation, displacement, or magnetothermal effects of magnetic nanoparticles to change local material states(Fig. 5B) [113]. Magnetic trilayer cartilage scaffolds establish gradient compression properties through the distribution of magnetic components in different regions. They can further produce tunable local deformation under an external magnetic field [114]. At the same time, magnetic fields can be used during manufacturing to control the spatial distribution of magnetic composite particles. Therefore, external fields can serve both as active driving tools during treatment and as spatial encoding tools during material preparation. Pulsed electromagnetic fields combined with cartilage materials represent a broader form of external physical regulation. Human cartilage tissue studies show that pulsed electromagnetic stimulation can increase proteoglycan synthesis and reduce some inflammation-induced catabolism [115]. Related three-dimensional construct studies and experiments combined with supramolecular hydrogels further show that electromagnetic stimulation can synergize with the material microenvironment(Fig. 5C) [116], [117]. Therefore, the core value of external physical driving is not to add functional labels such as ultrasound-responsive, magnetic-responsive, or electromagnetic-responsive. It is to give material mechanical instructions external switching, frequency setting, and dose adjustment capabilities. Future systems of this type need to pay more attention to spatial attenuation of physical fields in tissue, actual energy conversion efficiency within materials, and safety windows for long-term repeated stimulation.

**Fig. 5:**
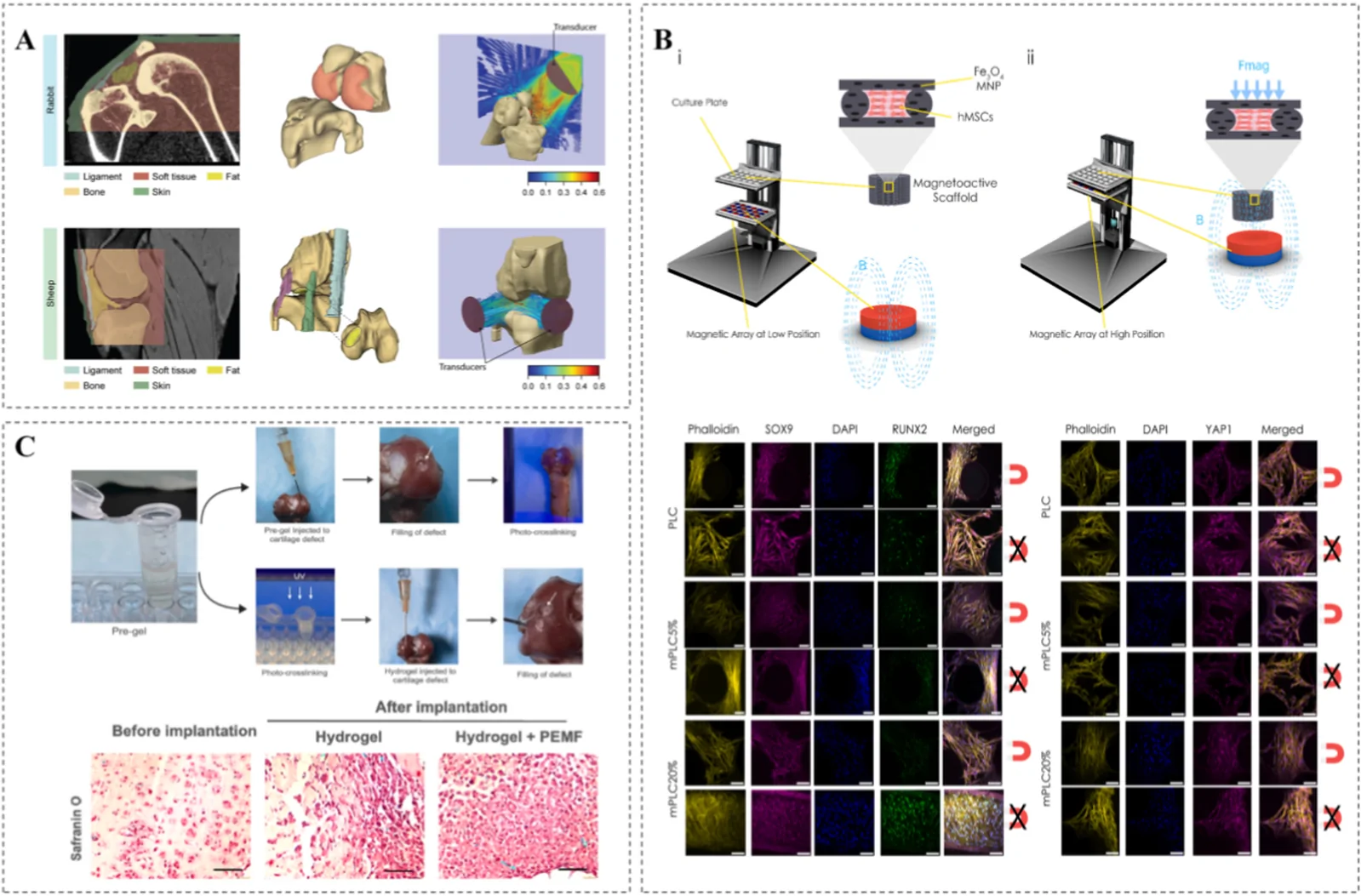
**Externally actuated mechanical stimulation for regulating cartilage mechanobiology** (A) Optimization of ultrasound stimulation parameters for piezoelectric hydrogel-mediated attenuation of osteoarthritis progression. Reproduced with permission from Ref. [112]. Copyright 2026, Elsevier; (B) Regulation of chondrogenic differentiation and mechanotransduction by 3D-printed scaffolds under different magnetic field strengths. Reproduced with permission from Ref. [113]. Copyright 2026, Elsevier; (C) Supramolecular hydrogel-mediated promotion of cartilage regeneration and repair under pulsed electromagnetic field stimulation. Reproduced with permission from Ref. [117]. Copyright 2023, Elsevier.

### 4.5 Mechanically coupled therapeutic delivery

Traditional intra-articular drug delivery mainly focuses on drug loading, release rate, and joint cavity retention. Mechanobiology-instructed delivery further requires that the mechanical state of the material directly participates in therapeutic output. Here, mechanical participation can mean that the material maintains stable drug release during repeated deformation. Mechanical participation can also mean that lubrication and drug release share the same interface. It can further mean that pressure, shear, or external physical stimulation directly regulates the release process [103]. First, a moving joint requires the drug-loaded network to have sufficient mechanical durability. If a soft material undergoes permanent rupture, fatigue, or rapid swelling during repeated compression, release kinetics will drift noticeably with movement state. Therefore, high toughness, self-recovery, and fatigue resistance are not engineering properties unrelated to drug delivery. They are important parameters determining whether local drug exposure is reproducible. Second, materials can integrate interfacial mechanical function with molecular therapy [118]. Hyaluronic acid microgels can simultaneously perform cartilage surface binding, highly hydrated lubrication, and disease-related responsive drug release. This allows interfacial shear and local drug exposure to be jointly determined by the same material structure [119]. Hyaluronic acid composite hydrogels also regulate lubrication and sustained release simultaneously through network hydration and structural stability [120]. The key to this design dimension is that mechanical function and drug function need to be complementary. It is not simply adding a drug to an existing hydrogel [121], [122]. Furthermore, mechanically triggered release allows physical input to directly enter therapeutic dose control. Tissue-adhesive hybrid hydrogel actuators have demonstrated that external deformation can change drug release rate. This converts mechanical input into chemical therapeutic output [123]. For joint applications, such designs especially need a reasonable trigger threshold. Normal walking, intense exercise, and pathological impact all cause material deformation. If release is simply linearly coupled to strain, premature depletion may occur under high activity. Mechanical-responsive delivery should also consider the reverse effect of drugs on material mechanics. The addition of drugs, nanoparticles, or protein factors may change crosslinking density, water absorption, network relaxation, and fatigue stability. Therefore, release curves and mechanical properties cannot be measured independently under two separate material conditions. Only joint evaluation under real drug-loaded states and repeated loading can determine whether mechanical therapy and pharmacological therapy truly coexist in the same system.

### 4.6 From mechanical matching to mechanical information programming

As mechanobiology-instructed materials accumulate more functions, classifying them mainly as hydrogels, scaffolds, microspheres, or nanoparticles no longer reflects their mechanical functions. The same hydrogel can simultaneously have stiffness gradients, stress relaxation, piezoelectricity, and drug delivery capacity. Different material forms can also achieve the same mechanical instructions. Therefore, a more explanatory classification is to organize materials according to how they process mechanical information(Fig. 6 and Table 1). Encoding corresponds to the baseline mechanical conditions preset before use. These include stiffness, porosity, anisotropy, surface structure, and spatial gradients. Adaptation corresponds to the material's ability to change over time. This includes viscoelasticity, stress relaxation, dynamic bond exchange, self-recovery, and mechanical evolution during degradation. Conversion corresponds to the transformation of mechanical energy into electrical potential, ion flow, or other physical signals directly readable by cells. Delivery corresponds to the material mechanical state participating in determining drug spatial localization, release duration, and stimulation-dependent output. These four levels are not independent. Static encoding determines how the material initially accepts and distributes load. Temporal adaptation determines how this load decays and reconstructs over seconds to weeks. Energy conversion determines whether mechanical input further generates new biophysical signals. Responsive delivery connects these physical changes with molecular therapy. Thus, the main line of material systems can be further summarized as a shift from mechanical matching to mechanical information programming. Materials not only need to resemble cartilage. They also need to receive, filter, convert, and output mechanical information in a controllable manner.

**Fig. 6:**
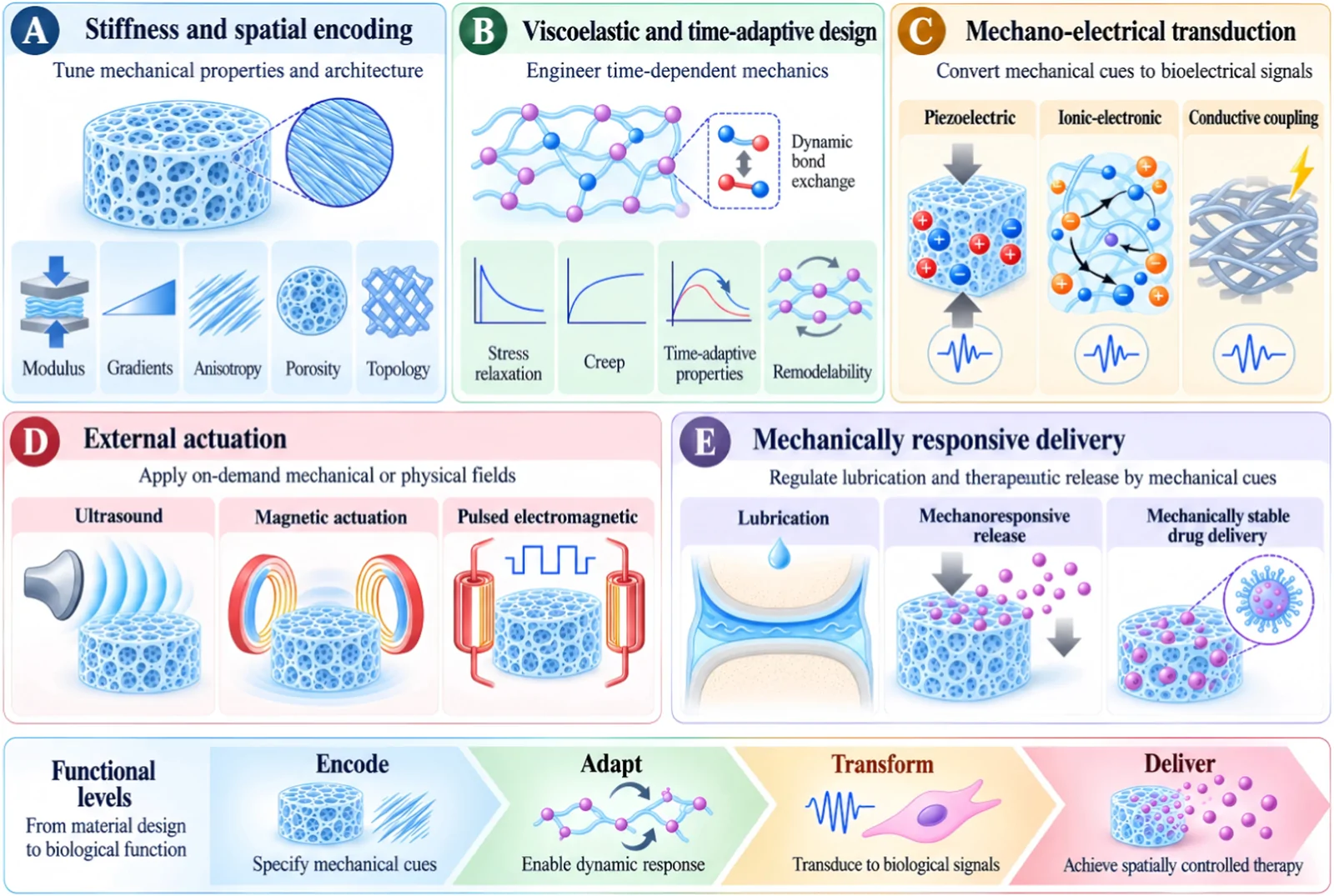
**Design dimensions of mechanobiology-instructed biomaterials.** (A) Stiffness, architecture, anisotropy, porosity, and spatial gradients. (B) Viscoelasticity, stress relaxation, dynamic bonds, and time-dependent remodeling. (C) Mechanoelectrical and mechanoionic signal conversion. (D) External physical actuation. (E) Mechanically responsive lubrication and therapeutic delivery.

**Table 1:** Functional design principles of mechanobiology-instructed biomaterials for osteoarthritis.

Functional dimension	Key material parameters	Design principle and mechanobiological function	Critical evaluation parameters	Representative References
Mechanical encoding	Stiffness, architecture, anisotropy, and spatial gradients	Program spatial resistance and load transfer to regulate cell- and tissue-level mechanical inputs	Local modulus, anisotropy, gradient continuity, and interfacial mechanics	[124], [125]
Temporal adaptation	Viscoelasticity, stress relaxation, creep, permeability, and dynamic crosslinks	Regulate time-dependent stress dissipation, network remodeling, and cellular mechanical exposure	Relaxation time, creep, permeability, cyclic recovery, hysteresis, and fatigue	[29], [126]
Endogenous mechanical energy conversion	Piezoelectricity, ionic mobility, conductivity, and mechanoionic coupling	Convert joint-motion-induced deformation into local electrical or ionic cues that modulate cellular responses	Activation threshold, input–output relationship, conversion efficiency, signal amplitude and frequency, wet-state stability, and cyclic durability	[106], [107], [108], [109]
Externally actuated instruction	Acoustic-, magnetic-, and electromagnetic-responsive components	Use external fields to regulate material and cellular states with controllable stimulation doses	Field intensity and frequency, penetration depth, spatial precision, conversion efficiency, dose–response relationship, and safety window	[112], [113], [127]
Mechanically coupled therapeutic delivery	Deformation responsiveness, adhesion, retention, and stimulus-dependent release	Couple mechanical protection or stimulation with local therapeutic delivery	Trigger threshold, loading-dependent release, retention, fatigue, and therapeutic durability	[118], [123]

## 5 Mechanobiology-instructed cell and tissue state remodeling

Mechanobiology-instructed biomaterials produce true biological effects only when cells can read their physical information and form stable state transitions.For OA, the key question is not whether a material increases a single cartilage marker. It is whether chondrocytes, stem cells, macrophages, synovial fibroblasts, subchondral bone cells, and senescent cells change their mechanical thresholds, cytoskeletal tension, metabolism, and long-term fate in a new mechanical environment. The same biomaterial parameters can be decoded differently by different cells. Therefore, there is no single path from biomaterial to tissue homeostasis. It is a multilayered mechanical response determined jointly by cell type, disease stage, and local tissue environment.

### 5.1 Chondrocyte mechanoadaptation and phenotypic homeostasis

The most direct mechanical fate of chondrocytes is not to produce more matrix [64]. It is to regain the ability to distinguish physiological from pathological load. Dynamic compression can promote homeostatic metabolism through TRPV4. An inflammatory environment can increase Piezo1 mechanosensitivity. This makes the same deformation produce stronger calcium signals and injury responses [52], [62], [128]. Thus, part of the chondrocyte state is actually a mechanical response threshold [129]. Material therapy needs to change not only matrix synthesis levels but also how cells interpret external load. Three-dimensional materials can reset this threshold by altering the cytoskeleton and spatial constraints. Higher sustained mechanical resistance usually enhances cell traction and cytoskeletal prestress. A more suitable local mechanical environment helps maintain round chondrocyte morphology and stable cartilage phenotype. Mechanical restriction studies further show that even without typical adhesion ligands, material restriction of cell volume expansion itself can form an independent mechanical input. Slow-relaxing networks restrict chondrocyte volume expansion and increase IL-1β secretion and catabolic genes. Fast relaxation allows cell expansion and more continuous cartilage matrix formation [30]. This result indicates that chondrocytes read materials not only through integrin adhesion. They also read volume restriction, osmotic changes, and sustained compression as three-dimensional specific mechanical processes [130]. Material stiffness further changes long-term nuclear state through the cytoskeleton and YAP [131]. Human osteoarthritic cartilage matrix stiffening is associated with increased YAP activity. When simulating different matrix stiffness, stiffer environments promote YAP nuclear localization and are accompanied by loss of cartilage phenotype. Chondrocyte-specific modulation of this pathway or local inhibitor delivery can reduce experimental degeneration [74]. The key here is not to simply define YAP as a pathological molecule. It is to show that materials can convert external mechanical environment into long-term transcriptional state by changing cytoskeletal tension. Time-dependent mechanical environment also determines whether chondrocytes can gradually escape sustained constraint. Dynamic networks release stress after cell traction. This allows newly formed collagen and glycosaminoglycans to expand beyond the pericellular region. Relatively fixed networks tend to form sustained local pressure. When cyclic compression is combined with different viscoelastic environments, the same macroscopic load is converted by the material into different cytoskeletal and nuclear mechanical inputs. Therefore, the core variables through which chondrocytes read mechanical instructions include membrane tension, cell volume, cytoskeletal prestress, local fluid movement, and nuclear mechanical state [130]. They are not the macroscopic modulus of the material itself. Mechanoelectrical instructions add another membrane electrophysiological layer. Local electrical potentials and ion migration generated by piezoelectric or ionoelectrical materials can further alter calcium influx and electrically sensitive signals. This allows cells to receive additional electrophysiological input under the same mechanical deformation. Thus, the ideal regulation of chondrocyte fate by biomaterials is not to continuously enhance anabolic metabolism. It is to help cells re-establish an appropriate load response window. This maintains adaptability during physiological activity and reduces excessive mechanical sensitization under high strain and inflammatory conditions.

### 5.2 Stem and progenitor cell fate across spatial and temporal mechanical niches

In cartilage tissue engineering, the selection and biological characteristics of seed cells are important determinants of regenerative outcomes [132]. Mesenchymal stromal cells and local progenitor cells are highly sensitive to the material mechanical environment. However, their fate is not determined by a single stiffness. Whether cells can spread, whether the local network allows remodeling, how long cytoskeletal tension is maintained, whether the spatial position is on the cartilage or bone side, and whether electromechanical stimulation is superimposed all jointly alter chondrogenic differentiation, osteogenic differentiation, and hypertrophic tendency [133]. Continuous stiffness gradients provide a direct platform to observe this spatial fate. Gradient particle scaffolds show that cells in soft regions tend to remain round and form glycosaminoglycan-rich matrix. Stiffer regions increase cell elongation, cytoskeletal tension, and mineralization-related expression. Inhibiting actin-myosin contraction markedly weakens this regional difference [134]. This result indicates that stem cells do not passively remain in different material regions. They actively read spatial gradients through cell traction and tension-dependent mechanotransduction. Dynamic gradients further make cell fate no longer completely determined by the material state at implantation. Alkaline phosphatase-responsive self-evolving hydrogels form differential secondary crosslinking according to local enzyme activity. This makes storage modulus change spatially and temporally with tissue state. It regulates migration and differentiation through integrin-related mechanical signals and PI3K/AKT/GSK-3β/β-catenin pathways [135]. Here, the biomaterial does not predefine final cell fate. It forms feedback with cells through continuously changing mechanical boundaries. Three-dimensional printed gradient scaffolds combined with programmed biomolecule delivery can integrate spatial structural cues with temporally controlled biochemical signals to regulate stem cell migration and chondrogenic/osteogenic differentiation for osteochondral regenerationt [124]. For cell fate, the importance of such systems is that mechanical and biochemical signals are not independent. Growth factors can determine what differentiation potential a cell has. Local stiffness, relaxation, and morphological constraints determine whether this potential is stably executed [99], [136]. Electromechanical signals can further alter cell migration and regional differentiation. Local electrical potential differences generated by cyclic loading can combine with material composition to form spatial instructions. This allows cells to simultaneously read mechanical resistance and electrophysiological environment. For osteochondral tissue, what truly needs to be avoided is cartilage-side progenitor cells entering hypertrophy and mineralization under long-term high tension, or bone-side cells failing to form effective load bearing in an overly soft environment. Therefore, the goal of mechanical fate regulation is not to promote more stem cell differentiation. It is to improve the spatial correctness and long-term stability of differentiation. This process also involves mechanical memory. Cytoskeletal tension and nuclear state formed in an early material environment may continue to affect subsequent differentiation even after the material degrades or local modulus changes [59], [137]. Therefore, the design of gradient and dynamic materials cannot focus only on implantation parameters. It also needs to consider the mechanical memory experienced by cells at different stages [126]. For stem cells, what materials truly change is the fate trajectory, not a differentiation marker at a single time point.

### 5.3 Mechanoregulation of synovial immune and fibrotic states

The osteoarthritic synovium does not simply receive inflammatory factors released by cartilage. Synovial matrix stiffness, tissue fibrosis, repeated stretching, and continuous joint surface wear can all alter the cytoskeletal state of macrophages and synovial fibroblasts. Therefore, the mechanical environment itself participates in maintaining immune phenotype. For these cells, the focus of material mechanical instructions is not to promote tissue formation [138]. It is to change inflammatory thresholds, migration, secretion, and fibrotic programs. Macrophages respond noticeably to material surface, local stiffness, and deformation [139]. When biomaterials reduce cartilage surface shear and damage, damage-associated molecular pattern release decreases. The sustained pro-inflammatory input to macrophages also decreases. At the same time, the material's own mechanical environment can affect macrophage adhesion morphology and cytoskeletal tension. Thus, macrophage mechanical fate is usually affected by both reduced external damage and direct material contact. Chondroitin sulfate nanoparticles combined with thermosensitive hydrogel for sustained fibroblast growth factor 18 delivery not only affect chondrocyte phenotype. They also promote a more anti-inflammatory macrophage state and reduce synovial inflammation [140]. Here, the material's effect on immune state does not come from a single anti-inflammatory drug. It comes from readjustment of the paracrine relationship between chondrocytes and macrophages after local microenvironment changes. Synovial fibroblasts lie at the intersection of mechanical and metabolic signals. Baicalin functional polymer integrated into a dynamic Schiff base network improves material self-repair and lubrication. It can also enter synovial fibroblasts and regulate YAP1 and glycolytic state. This reduces secretion of inflammatory mediators such as IL-1β, IL-6, and IL-8 [141]. This indicates that synovial fibroblast reading of the mechanical environment can further connect energy metabolism and inflammatory secretion. Biomaterials can promote state transition by changing local tension and molecular signals together. Neuroimmune cells may also participate in this process. Thermosensitive hydrogels with sustained anti-inflammatory peptide release can regulate resident macrophage states in the dorsal root ganglion and reduce persistent pain [142]. Although this action mainly involves neuroimmune regulation, it suggests that the cell ecosystem remodeled by mechanical materials is not limited to the cartilage surface. It may change joint usage and mechanical load through pain, activity level, and inflammatory state. Therefore, mechanical immune regulation is better understood as a cell state network, not a single material function [131]. Macrophages and synovial fibroblasts read combined information from local matrix tension, damage degree, cytokines, and metabolic environment. If a material can reduce abnormal mechanical input and simultaneously attenuate inflammatory metabolic programs, it may shift the synovium from a continuously amplifying state toward a more stable immune environment.

### 5.4 Subchondral bone mechanoadaptation and osteochondral load transfer

Subchondral bone cells have different mechanical tasks from chondrocytes [143]. Osteoblasts, osteocytes, and osteoclasts need to convert local stress changes into bone formation, bone resorption, and microstructural adjustment [144]. Their mechanosensing is therefore part of tissue adaptation. The problem in OA is not that subchondral bone has a mechanical response. It is that this response forms excessive or spatially uneven remodeling under sustained abnormal load. This further alters the mechanical boundary of overlying cartilage. Human osteoarthritic subchondral bone and cell studies show that FOXC1 and Piezo1 are elevated and linked to YAP-related osteogenic states [48]. Animal and cell studies further show that abnormal load can promote abnormal remodeling through osteoblast Piezo1, intracellular calcium, and JAK2/STAT3 pathways. This is associated with uneven tibial plateau depression [49]. These findings indicate that subchondral bone cells do not merely bear altered loads secondary to cartilage degeneration. They actively convert mechanical abnormalities into structural and molecular changes that can feed back to cartilage. Recent evidence extends this concept from local bone remodeling to direct bone-to-cartilage communication. Mechanical stress can stimulate subchondral osteocytes to release extracellular vesicles enriched in miR-23b-3p, which subsequently alter OTUD4-dependent mitophagy and metabolic homeostasis in chondrocytes, thereby promoting cartilage catabolism and OA progression [145]. Thus, deep-tissue mechanosensing can be propagated across the osteochondral unit through both mechanical boundary changes and intercellular molecular communication. The effect of materials on these cells first comes from changing load boundaries. Continuous stiffness or mineralization gradients can reduce abrupt stress transfer between cartilage and bone. This allows cells at different depths to experience a mechanical range closer to their own function [92]. For bone-side cells, the material does not simply provide a stiffer environment. It needs to provide mechanical conditions sufficient for osteogenesis and load bearing without causing local excessive stress concentration. Regional materials can also change bone-side cell fate through cytoskeletal and mechanosensitive transcriptional states. Soft regions favor a cartilage-like state. Stiffer and mineralized regions increase traction and osteogenic phenotype. If this difference exists as a continuous gradient, cells can form a regionalized state from cartilage to bone [96], [146]. They do not suddenly switch on either side of a discrete interface. Therefore, the goal of subchondral bone cell mechanical fate is not to uniformly inhibit osteogenesis. It is to re-establish bone remodeling matched to load. What materials need to change is the stress amplitude, spatial distribution, and interface continuity sensed by deep cells. This prevents subchondral bone from responding to joint load through continuous sclerosis and abnormal bone formation.

### 5.5 Aging and metabolic constraints on mechanoadaptation

Whether cells can adapt to the mechanical environment depends heavily on their metabolism and mitochondrial state. Sustained abnormal load can cause calcium homeostasis disturbance, reactive oxygen species accumulation, mitochondrial dysfunction, and cellular senescence [128], [147]. Senescent cells further release inflammatory and matrix-degrading factors through the senescence-associated secretory phenotype. Thus, aging and metabolism are not independent modules outside mechanobiology. They are important intrinsic conditions determining whether cells can restore mechanical homeostasis. High-fluidity hydrogel microspheres use deformable liposomes as dynamic crosslinking cores. This improves network deformation and stress dispersion. At the same time, rolling lubrication reduces local mechanical stress. This is accompanied by improved mitochondrial function and reduced chondrocyte senescence(Fig. 7A) [148]. The importance of such research is linking material mechanical buffering to cellular senescence state. It shows that reducing abnormal stress can change long-term cell fate, not just provide short-term membrane protection. More direct mitochondrial regulation uses cartilage-targeted interpenetrating networks to deliver mitochondria-directed manganese dioxide nanoparticles. It simultaneously provides oxygen and nicotinamide adenine dinucleotide precursors. This improves respiratory chain electron donor-acceptor balance and redox state and reduces the senescence-associated secretory phenotype [149]. This result suggests that mechanical adaptation requires sufficient energy support. Chondrocytes depend on mitochondrial function to maintain ion balance, cytoskeletal remodeling, and matrix synthesis [150]. The Gel@CAT-L system targeting mitochondrial protein homeostasis can reduce reactive oxygen species and maintain mitochondrial homeostasis through Sirtuin 3-related mitochondrial unfolded protein response [151]. ROS-responsive DNA hydrogels reduce chondrocyte senescence through free radical scavenging and regulation of NF-κB and mammalian target of rapamycin signaling [152]. These studies show that cell fate recovery depends not only on how much a material reduces external load. It also depends on whether cells still have sufficient metabolic capacity to re-enter homeostasis. There is also a direct link between mechanical and endoplasmic reticulum stress. Hydrogel microspheres with tunable stress relaxation can reduce chondrocyte endoplasmic reticulum stress by dissipating mechanical vibration. Simultaneous delivery of tauroursodeoxycholic acid further reduces related stress responses and delays OA progression(Fig. 7B) [101]. This study forms a more direct connection between dynamic mechanical buffering, cellular stress, and long-term fate. It also suggests that aged or metabolically impaired cells may require combined intervention of mechanical protection and organelle homeostasis regulation. Therefore, aging-metabolic cells are not simply less responsive to materials. They may read the same mechanical instruction in different ways. For such cells, simply restoring normal modulus or loading may not be enough to establish homeostasis. It is necessary to simultaneously reduce mechanical burden and restore mitochondrial, redox, and protein homeostasis. This allows cells to regain the ability to adapt to the external mechanical environment (Fig. 5A).

**Fig. 7:**
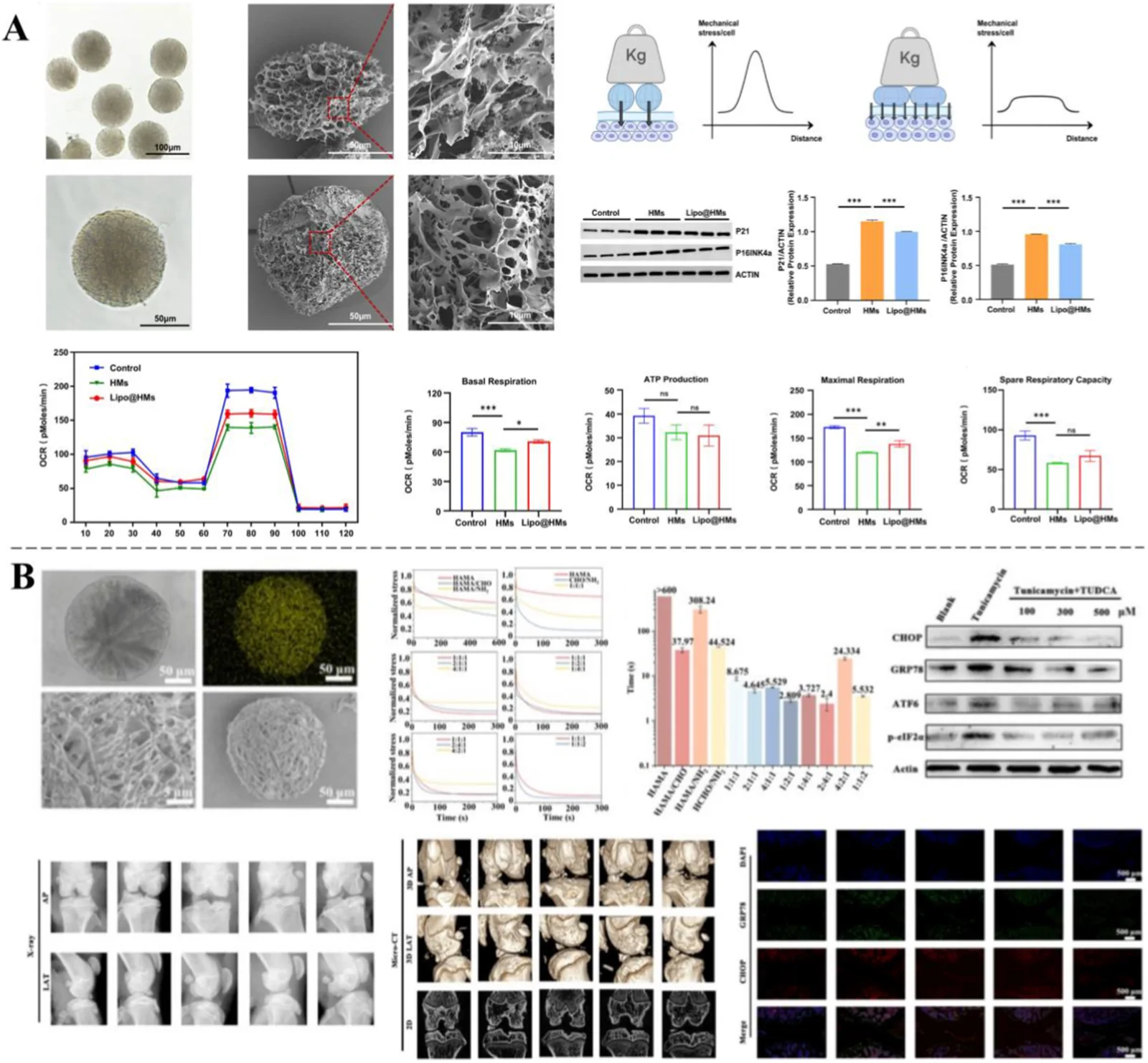
**Dynamic stress-dissipating biomaterials alleviate mechanical stress and restore cellular homeostasis on mechanoadaptation in osteoarthritis** (A) High-mobility-network hydrogel microspheres provide joint lubrication and reduce chondrocyte mechanical stress, thereby attenuating osteoarthritis through regulation of cellular senescence and mitochondrial function. Reproduced with permission from Ref. [148]. Copyright 2025, Elsevier. (B) Viscoelastic drug-loaded hydrogel microspheres dissipate joint mechanical stress and modulate cellular stress responses to delay osteoarthritis progression. Reproduced with permission from Ref. [101]. Copyright 2025, SPJ.

### 5.6 From cell-state remodeling to whole-joint homeostasis

Improvement in a single cell state does not automatically translate into whole-joint mechanical homeostasis. Chondrocytes, synovial macrophages and fibroblasts, subchondral bone cells, and sensory nerves continuously interact through mechanical and molecular signals [145], [153]. Therefore, local cell fate changes caused by biomaterials further alter the mechanical environment of other tissues [131]. Recovery of chondrocyte state can increase matrix synthesis and local load bearing. This makes tissue load distribution more uniform. After cartilage surface damage decreases, the damage-associated molecules and inflammatory stimuli received by the synovium also decrease. In turn, reduced synovial inflammation can weaken inflammatory sensitization of mechanosensitive systems such as Piezo1 [138]. This allows chondrocytes to regain a more reasonable mechanical threshold. Here, there is a closed loop from mechanical environment to cell state, and then from cell state back to mechanical environment [139]. Subchondral bone also participates in this feedback. When abnormal deep bone remodeling is alleviated, the boundary stiffness and local stress distribution of overlying cartilage change accordingly [145]. If subchondral bone continues to sclerose, even if chondrocytes locally obtain a suitable material environment, the overall joint load may still re-concentrate. Thus, chondrocyte and subchondral bone cell fates cannot be understood separately. The concept of mechanical and metabolic dual gradients further suggests that coupling between different tissues does not depend only on stiffness. Oxygen tension, vascular environment, nutrient exchange, and metabolic state may all combine with mechanical gradients to determine regional cell phenotypes [154]. Therefore, multi-tissue mechanical coupling does not require all tissues to return to the same mechanical parameter. It requires each cell type to be in a mechanical and metabolic range matching its function (Fig. 8). From the perspective of cell fate, mechanobiology-instructed materials ultimately need to coordinate multiple levels. Chondrocytes restore mechanical tolerance and matrix homeostasis. Stem cells form spatially correct and long-term stable differentiation. Synovial immune cells no longer continuously amplify inflammation. Subchondral bone cells re-form load-matched remodeling. Senescent cells regain sufficient metabolic adaptability. Only when these states gradually become complementary can the local mechanical instructions provided by materials translate into whole-joint functional recovery.

**Fig. 8:**
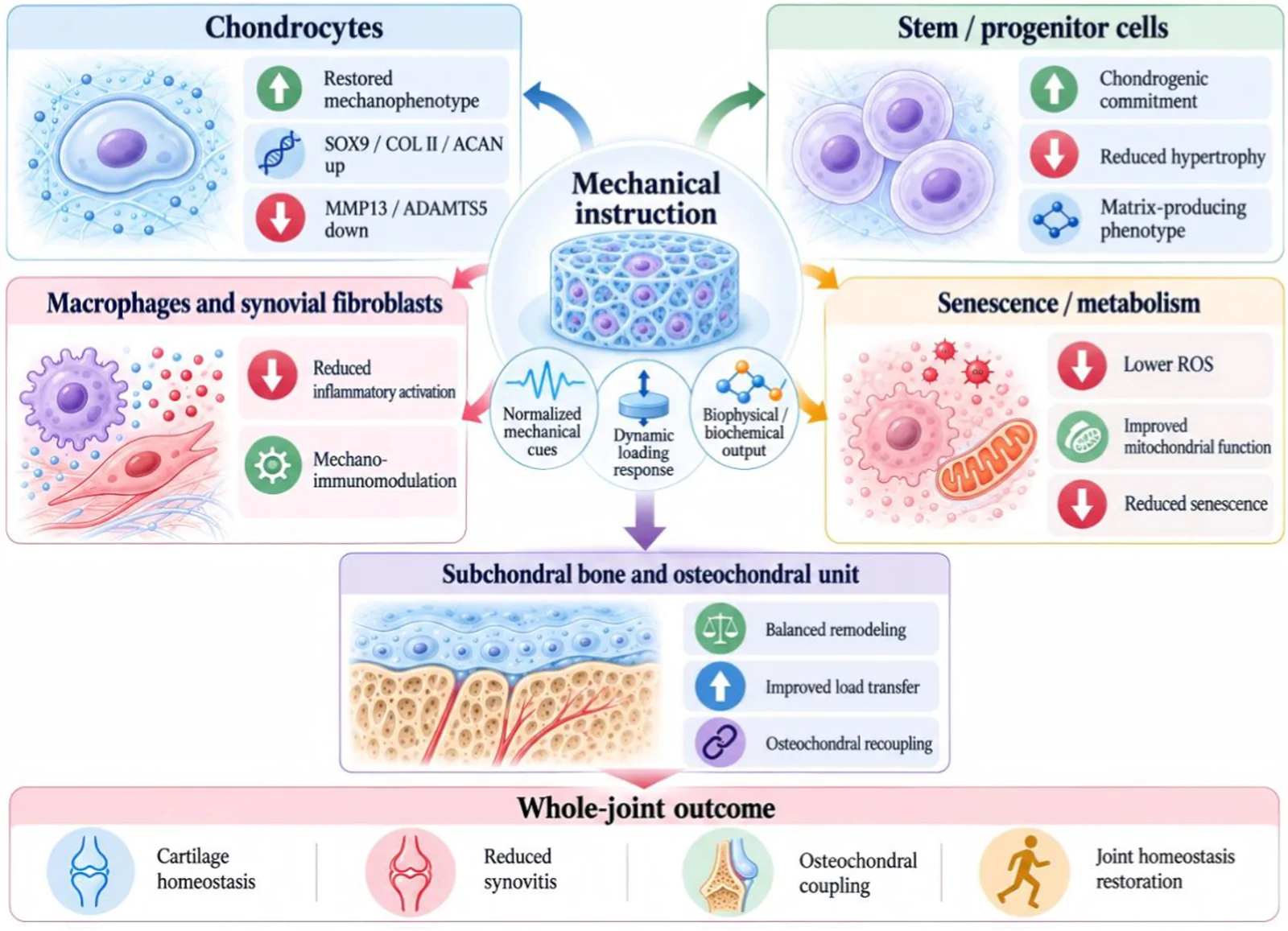
**Mechanobiology-instructed biomaterials regulate cell and tissue states toward whole-joint homeostasis.** Material-mediated mechanical cues and downstream biological responses influence chondrocyte phenotype, stem/progenitor cell chondrogenesis, synovial inflammation, mitochondrial function, cellular senescence, and osteochondral remodeling.

## 6 Phenotype-guided mechanobiological intervention for OA disease modification

The key to whether mechanobiology-instructed materials truly show disease-modifying potential is not how many functions the material itself has, but whether these functions address the dominant pathological mechanical abnormalities in a patient or model. Importantly, the phenotypes proposed here represent analytical priorities for mechanobiological intervention rather than mutually exclusive or clinically validated OA subtypes. Individual joints often exhibit overlapping pathological features, including lubrication failure, load instability, inflammatory mechanosensitization, subchondral bone remodeling, and aging-related metabolic dysfunction. The relative contributions of these abnormalities may also change during disease progression. Accordingly, this section discusses how material functions can be prioritized according to the prevailing mechanical pathology and which structural, mechanical, and functional endpoints are needed to evaluate their disease-modifying potential.

### 6.1 Friction-dominant phenotype: interfacial lubrication and surface protection

When cartilage surface lubrication decreases, roughness increases, or superficial matrix already shows early damage, friction itself can become a sustained pathological input. High friction increases superficial shear strain [155], [156], [157]. This causes the cartilage surface to repeatedly experience local damage during each joint movement. Therefore, the most direct disease-modifying logic for this phenotype is to prioritize reducing interfacial shear, rather than first increasing deep material stiffness. Highly hydrated biomaterials can form a stable hydration layer on the cartilage surface [121]. This keeps boundary lubrication at low friction even under high load. Li et al. developed a cartilage-mimetic lubricating hydrogel using a highly hydrated network and interfacial hydration to achieve good tribological performance [158]. Huang et al. further combined surface hydration with underlying mechanical support through a cartilage-mimetic multilayer hydrogel coating. This improved lubrication layer stability under continuous aqueous environment and repeated friction [159]. Such studies show that the therapeutic focus for the high-friction phenotype is not simply increasing viscosity. It is to establish an interfacial hydration structure that persists under repeated shear [160], [161], [162]. Early lesions are often focal. Therefore, whether materials can reach the actual high-friction region is equally important. Targeted hydrogel microspheres use type I collagen exposed on damaged cartilage surfaces as binding sites. This allows highly hydrated microspheres to preferentially accumulate in local lesions and form a lubricating layer. Animal experiments show reduced early cartilage degeneration [163]. The significance of spatial targeting is to increase effective lubricating material concentration per lesion area. It also reduces ineffective distribution and rapid clearance throughout the joint cavity. For this phenotype, evaluation endpoints should directly correspond to the mechanism of action. In addition to histological scoring, it is more important to observe friction coefficient, lubricating layer duration, wear after repeated shear, superficial cartilage strain, and surface coverage stability in a moving joint. If a material only improves staining without proving long-term friction reduction, it is difficult to judge whether it truly changes high-friction pathology. High-friction phenotypes often coexist with inflammation. Therefore, lubrication does not exclude combination therapy [122], [164]. If the main contradiction remains surface mechanical damage, lubrication can serve as upstream foundational intervention. Anti-inflammatory or antioxidant modules can then be added [118], [165], [166].

### 6.2 Load-instability phenotype: dynamic buffering and load redistribution

Post-traumatic OA, ligament injury, joint instability, and obvious malalignment often have abnormal load amplitude, direction, or temporal distribution as the main driving force [167], [168], [169]. In such cases, even if a biomaterial has suitable static modulus, it may not handle repeated impact, local high strain, and abnormal load transfer. Therefore, the priority should shift from matching stiffness to redistributing and dissipating dynamic load. A moving joint first requires materials to maintain structure under continuous compression and shear. Joshi et al. constructed a mechanically tough and soft hydrogel. It can recover rapidly under simulated high-activity mechanical conditions and maintain sustained release of cathepsin K inhibitor. It reduced cartilage degeneration in a post-traumatic OA model [103]. The significance of this study for dynamic mechanical abnormality-type OA is that softness and mechanical durability can coexist. Materials do not need to resist joint load simply by increasing modulus. Cell-shock-absorbing hydrogel microspheres with tunable stress relaxation further directly target mechanical vibration and impact. The material adjusts network relaxation time through oxidation and hydrazone coupling. This allows microspheres to dissipate mechanical stimulation. Combined with tauroursodeoxycholic acid, it reduces chondrocyte endoplasmic reticulum stress and delays OA progression in animal models [101]. Here, the core of disease intervention is not to promote more tissue formation. It is to reduce the efficiency of abnormal mechanical energy transfer to cellular stress. Joint instability or malalignment also involves load redistribution at the whole-joint scale. Local injectable materials can buffer part of the impact. However, they cannot automatically correct persistent kinematic abnormalities. Therefore, for this phenotype, material studies should simultaneously evaluate gait, load distribution, contact area, and local strain [170], [171], [172]. They should not rely only on cartilage histology. If abnormal kinematics persist, materials are more suitable as mechanical buffering and local protection. They are not a replacement for stability reconstruction. Dynamic mechanical abnormality phenotypes also require attention to mechanical evolution during material degradation. If a material can buffer load early but then undergoes large drift in relaxation time and modulus after weeks due to water absorption, degradation, or fatigue, its disease-modifying effect may also disappear. Therefore, long-term cyclic fatigue, stress relaxation retention, and load transfer after degradation should become important evaluation endpoints for this phenotype.

### 6.3 Mechanosensitized phenotype: normalization of mechanotransduction

Some OA is not only about excessive external load. It involves excessive cellular response to normal or moderate load [64]. Inflammation-induced Piezo1 sensitization, matrix stiffening promoting YAP nuclear translocation, and long-term high cytoskeletal tension can all abnormally amplify mechanical signals [128]. For this phenotype, simply reducing joint load may not be enough to restore cell state. Material therapy needs to further target mechanotransduction itself. Human osteoarthritic cartilage matrix stiffening is associated with increased YAP activity. Matrices simulating different stiffness show that stiff environments promote YAP nuclear localization and loss of cartilage phenotype. Chondrocyte-specific modulation of this pathway or local verteporfin delivery can reduce experimental cartilage degeneration [74]. This study shows that mechanotransduction abnormality-type therapy can directly target the intracellular amplification step of pathological mechanical signals. It does not have to change only the macroscopic load of the entire joint. Mechanoelectrical materials provide another way to reprogram mechanotransduction [127]. After mechanical input is converted by the material into controllable electrical signals, it can bypass part of the abnormal matrix force transmission process. This allows cells to receive new physical stimulation through membrane electrical and calcium signals. Its potential advantage is reorganizing mechanical information rather than simply reducing all mechanical input. However, this strategy also requires clear dosing because mechanically sensitized cells may have different thresholds for electromechanical stimulation. Mechanotransduction abnormality phenotypes especially require rigorous mechanistic validation. If Piezo1, FAK, or YAP expression changes after material treatment, this does not automatically prove that these changes mediate disease modification. More ideal studies need to combine pathway inhibition, gene knockdown, or rescue experiments [173], [174]. They should determine whether the material effect disappears or noticeably weakens after blocking key mechanical nodes. From a therapeutic principle perspective, this phenotype is better suited to normalization rather than shutting down mechanical signals. Pathways such as TRPV4 participate in physiological dynamic load responses [175], [176]. YAP also has cell type- and stage-dependent roles [177], [178]. Therefore, continuous comprehensive inhibition may disrupt normal tissue adaptation. The goal of disease modification should be to adjust the mechanical sensitivity threshold back to a range that can distinguish physiological from pathological load.

### 6.4 Inflammation–mechanics coupling: breaking the pathological feedback loop

Inflammation-sensitized OA is characterized by mutual amplification between mechanical abnormality and inflammation [179]. Inflammatory mediators can increase mechanosensitive channel expression and responsiveness. This makes the same mechanical deformation produce stronger injury [52], [180]. Sustained friction and strain can further promote tissue damage and inflammatory activation, creating a positive feedback between abnormal mechanics and inflammation. Therefore, lubrication alone may not reverse an established inflammatory program, whereas anti-inflammatory therapy may provide only limited protection if continuous mechanical damage persists. Mechanical protection and inflammatory regulation should therefore be coordinated for this phenotype. Recent biomaterial studies provide several approaches to this coupled intervention. A biomimetic lubricating nanosystem based on hyaluronic acid-modified fluorinated graphene combines sustained interfacial lubrication with stimuli-responsive drug release, simultaneously reducing friction and suppressing inflammatory and catabolic responses in OA model [181]. Similarly, a double-crosslinked self-healing hydrogel provides protection against cartilage wear while regulating NF-κB inflammatory signaling, illustrating how mechanical surface protection and molecular inflammatory regulation can be incorporated within the same therapeutic system [182]. More recently, lymphocyte-derived engineered apoptotic bodies with cartilage affinity were incorporated into lubricating hydrogel microspheres. In this system, the microspheres reduce cartilage-surface friction and restore local mechanical conditions, while the engineered apoptotic bodies regulate the inflammatory microenvironment and support cartilage homeostasis [183].These studies suggest that mechanical and biochemical functions do not need to operate as independent therapeutic modules, instead, reduction of pathological mechanical input can be spatially and temporally coupled with regulation of the established inflammatory state. For the inflammation–mechanics coupling phenotype, this coordination is particularly relevant because mechanical damage and inflammatory activation frequently coexist within the same joint environment. A biomaterial that simultaneously reduces local friction or mechanical injury and suppresses inflammatory sensitization may therefore be better positioned to interrupt the pathological feedback than either intervention alone. Inflammation-sensitized phenotypes may also involve persistent activation of synovial fibroblasts and macrophages. Consequently, evaluation should extend beyond cartilage histology to include synovial thickness, macrophage phenotype, inflammatory mediators, pain-related behavior, and cartilage mechanosensitive pathways together with relevant mechanical outcomes. Only when both pathological mechanical input and inflammatory amplification are improved can a material be considered to act on this coupled disease process. This framework is also relevant to obesity-associated OA [184]. Obesity increases joint loading while simultaneously promoting metabolic inflammation and altered adipokine signaling [185], [186]. Thus, the dominant pathology may involve both excessive mechanical demand and inflammatory-metabolic sensitization. In such cases, the relative contributions of local friction, abnormal load distribution, synovial inflammation, and systemic metabolic disturbance should first be identified, providing a pathological basis for determining the balance between mechanical protection and molecular therapy.

### 6.5 Osteochondral interface phenotype: restoration of continuous load transfer

When subchondral bone sclerosis, trabecular remodeling, and osteochondral interface stiffness mismatch become the main abnormalities, adding a highly hydrated material only to the cartilage surface cannot correct deep load. The therapeutic focus for this phenotype should shift from surface protection to continuous load transfer across the osteochondral interface [187], [188]. At the same time, it should avoid new stress concentration caused by an overly soft upper cartilage layer and an overly hard lower bone layer. Three-dimensional printed gradient composite scaffolds can form differential tissue states in different regions through composition and programmed biomolecule release [124]. Phosphate glass-reinforced acrylamide gradient systems establish a continuous mechanical change from soft to hard. This allows load to gradually transfer over a larger spatial range [125]. Double-layer hydrogels with directional microchannels and mechanical gradients further balance regional load bearing, cell migration, and mass transport [189]. Advanced photopolymerization three-dimensional printing further improves spatial control of composition, porosity, and mechanical gradients [190]. For disease modification, the value of these technologies is not only producing a cartilage layer and a bone layer. It is reducing mechanical mismatch between soft and hard tissues. This allows the material to maintain reasonable load transition during tissue maturation and degradation. Subchondral bone cells themselves are mechanosensitive. Piezo1 and related calcium signals participate in pathological osteogenesis driven by abnormal load [191]. Therefore, restoring interface mechanics may not only improve tissue-level stress. It may further alter mechanotransduction in deep cells. For OA with obvious subchondral bone sclerosis, more reasonable disease endpoints include subchondral bone plate thickness, trabecular parameters, interface stress transfer, and local strain in overlying cartilage. Efficacy cannot be judged only by cartilage staining. Therefore, when imaging or histology indicates obvious subchondral bone sclerosis and interface abnormality, gradient or osteochondral interface strategies should theoretically have higher priority. However, specific design still needs to distinguish whether the main problem is excessive sclerosis, bone loss, or local depression. Different deep lesions correspond to different mechanical boundaries.

### 6.6 Aging–metabolic phenotype: rebalancing mechanical demand and adaptive capacity

Age-related OA and some forms of long-term chronic OA involve not only deterioration of the extracellular mechanical environment but also a progressive decline in the intrinsic capacity of chondrocytes to adapt to mechanical stress [35], [192], [193]. Abnormal mechanical loading can itself accelerate this transition. Mechanical stress activates Piezo1-dependent Ca^2+^ signaling and promotes chondrocyte senescence and senescence-associated inflammatory responses [147]. Therefore, aging and mechanical overload should not be regarded as independent drivers. They can converge on cellular states that progressively narrow the range of mechanical conditions that cartilage can tolerate. Mitochondrial dysfunction is an important link between mechanical stress, aging, and cellular senescence. Mechanical overload and aging reduce PDZK1 expression in chondrocytes, whereas PDZK1 deficiency promotes mitochondrial damage, reactive oxygen species accumulation, and senescence, ultimately aggravating OA progression [194]. Mechanosensitive signaling can further amplify mitochondrial injury. Elevated Piezo1 activity increases intracellular Ca^2+^ loading and mitochondrial permeability, promoting mitochondrial DNA release and cGAS-STING activation in chondrocytes [150]. Thus, even when external mechanical burden is reduced, established mitochondrial dysfunction and senescence may continue to maintain catabolic signaling. The mechanical microenvironment surrounding aged cells also changes. The pericellular matrix normally filters and transmits mechanical signals to chondrocytes, whereas its enzymatic degradation alters local biomechanical properties and disrupts mechanotransduction in OA [130]. Consequently, reproducing the bulk modulus of young cartilage alone may not restore an appropriate cellular mechanical environment. Materials with suitable stress relaxation, impact dissipation, and lubrication may instead reduce the mechanical demand imposed on metabolically compromised cells. Metabolic reprogramming provides another layer of this phenotype. TXNIP-associated changes in glucose metabolism are linked to mitochondrial dysfunction, oxidative stress, inflammation, and impaired extracellular matrix homeostasis in OA chondrocytes [195]. Lipid metabolism is also directly involved: excessive chondrocyte fatty acid oxidation increases acetyl-CoA availability, alters protein acetylation and SOX9 stability, and promotes OA progression [196]. These findings are particularly relevant to obesity- and metabolic syndrome-associated OA, in which increased mechanical loading may coexist with systemic and local metabolic inflammation. Therefore, disease modification for the aging-metabolic phenotype should not rely on mechanical unloading alone. Mechanical outcomes such as gait, joint loading, and local strain should be evaluated together with mitochondrial function, reactive oxygen species, senescence-associated markers, and glucose or lipid metabolism. In obesity-related models, adiposity and synovial inflammation should also be considered. Mechanobiology-instructed materials should ultimately reduce excessive mechanical demand while metabolic, mitochondrial, or anti-senescence regulation restores the intrinsic capacity of cells to tolerate and appropriately respond to mechanical stimulation.

### 6.7 From phenotype matching to disease-modifying evaluation

Given the overlapping and evolving nature of OA mechanical pathology, phenotype-guided intervention should be regarded as a flexible framework for prioritizing therapeutic targets rather than a fixed classification system [197], [198], [199].When high friction and superficial surface damage dominate, the priority should be restoring boundary lubrication and lesion area coverage. Friction, shear, and surface durability should be the main mechanical endpoints. When post-traumatic joint instability, abnormal alignment, or impact load dominates, load redistribution, dynamic buffering, and fatigue stability should be prioritized. Gait, load bearing, and local strain should be included in evaluation. When subchondral bone sclerosis and osteochondral interface abnormality are obvious, gradient and osteochondral interface designs have more direct pathological correspondence. Bone remodeling and cross-interface load should be observed simultaneously. When inflammatory sensitization is obvious, mechanical protection alone is often insufficient [179]. It is more suitable to combine lubrication or dynamic unloading with anti-inflammatory modules. When aging, mitochondrial damage, and senescence-associated secretory phenotype dominate, dynamic mechanical unloading should be combined with mitochondrial, oxidative stress, or anti-aging interventions (Fig. 9 and Table 2).

**Fig. 9:**
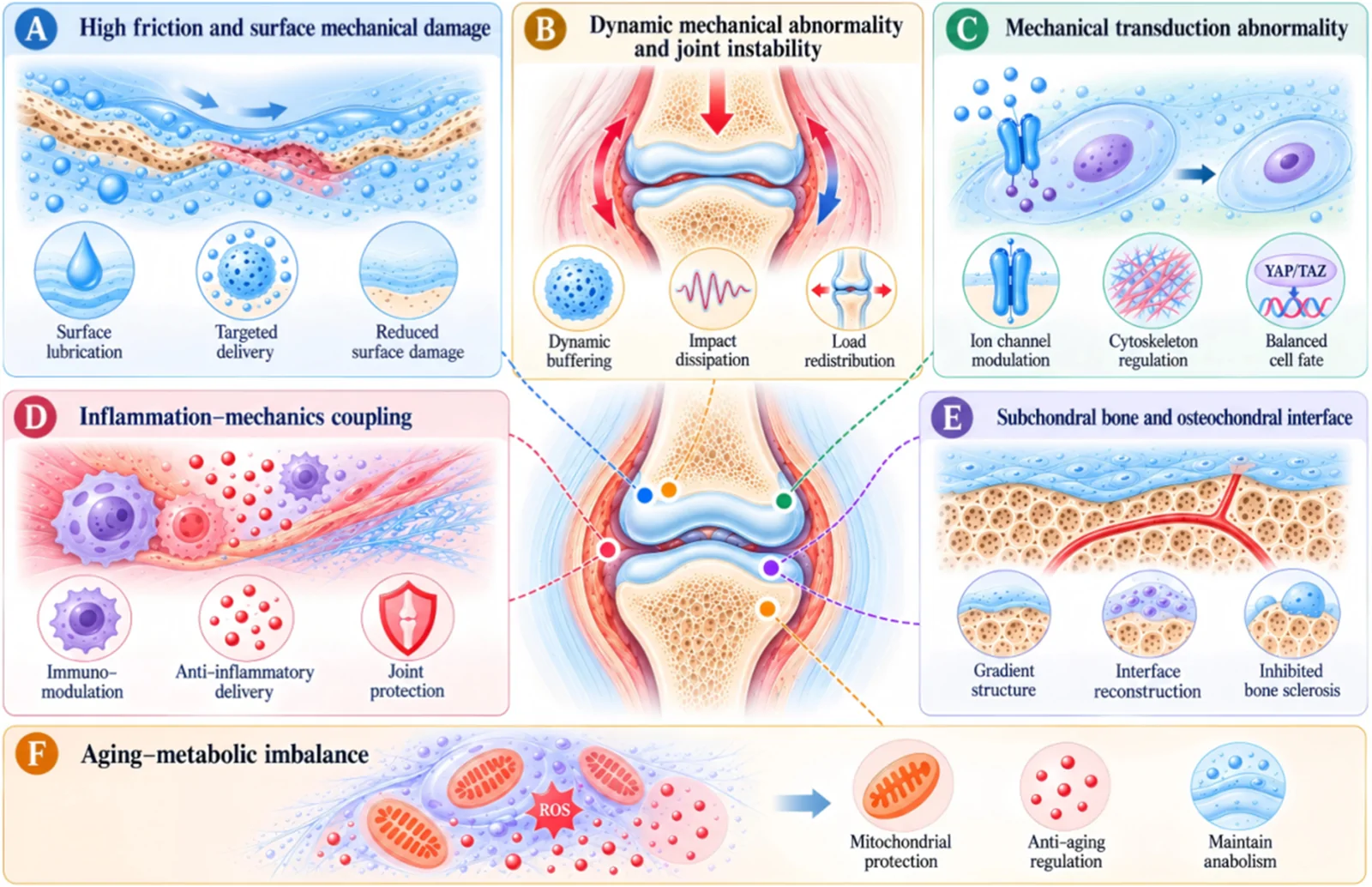
**Phenotype-guided mechanobiological intervention strategies for osteoarthritis.** (A) Friction-dominant pathology: restore interfacial lubrication and reduce shear. (B) Load instability: buffer and redistribute excessive mechanical loads. (C) Mechanotransduction dysregulation: normalize cellular mechanical sensitivity and signaling thresholds. (D) Inflammation–mechanics coupling: combine mechanical protection with control of inflammatory amplification. (E) Osteochondral interface abnormalities: restore continuous load transfer. (F) Aging-related metabolic dysfunction: reduce mechanical demand and support mitochondrial and cellular adaptation.

**Table 2:** Phenotype-guided mechanobiological intervention and disease-modifying evaluation in osteoarthritis.

OA mechanical phenotype	Dominant mechanical pathology	Therapeutic objective	Mechanobiological intervention principle	Mechanistic validation	Disease-modifying endpoints	Representative references
Friction-dominant	Lubrication failure, elevated surface friction, repetitive shear, and superficial wear	Restore low-friction articulation and prevent repetitive surface injury	Reduce interfacial friction and shear while maintaining durable surface protection under repeated articulation	Demonstrate sustained reduction of friction and shear under physiologically relevant cyclic loading	Friction coefficient, wear, superficial strain, surface integrity, retention, and durability	[157], [159], [163]
Load-instability/PTOA	Impact, instability, heterogeneous load redistribution, and repetitive high strain	Reduce transmission of pathological mechanical energy	Redistribute local loads and dissipate transient mechanical energy without excessive stress shielding	Demonstrate reduced tissue/cellular stress or strain during dynamic loading	Gait, contact mechanics, local strain, cyclic fatigue, and cartilage degeneration	[103], [168], [169]
Mechanosensitized	Excessive cellular response to otherwise tolerable mechanical input	Restore the physiological mechanosensitivity window	Normalize the magnitude, duration, and cellular decoding of mechanical inputs rather than globally suppress mechanotransduction	Combine cellular mechanical measurements with genetic, pharmacological, or rescue-based pathway validation	Mechanical-response threshold, Ca^2+^ signaling, cytoskeletal/nuclear state, and cartilage homeostasis under loading	[52], [128], [130], [147]
Inflammation–mechanics coupled	Mechanical injury sustains inflammatory activation, whereas inflammation enhances mechanosensitivity	Break the bidirectional pathological feedback loop	Concurrently attenuate pathological mechanical input and inflammation-induced mechanical sensitization	Demonstrate simultaneous attenuation of mechanical injury and inflammatory sensitization	Friction/load, synovitis, inflammatory cell state, inflammatory mediators, pain, and cartilage preservation	[181], [182], [183]
Osteochondral interface-dominant	Subchondral sclerosis, mechanical mismatch, and disrupted cartilage–bone load transfer	Restore continuous osteochondral load transmission	Re-establish spatially continuous mechanical transitions and coordinated cartilage–bone load sharing	Demonstrate normalization of interface mechanics rather than cartilage formation alone	Interface stress/strain, cartilage mechanics, subchondral microarchitecture, and osteochondral integrity	[124], [125], [143]
Aging–metabolic	Reduced cellular adaptive capacity despite persistent mechanical demand	Rebalance mechanical demand and cellular adaptive capacity	Reduce mechanical demand while restoring mitochondrial, metabolic, and stress-adaptive capacity	Demonstrate reduced mechanical burden together with restored cellular stress adaptation	Loading/gait, mitochondrial function, ROS, senescence/metabolic state, and sustained cartilage protection	[147], [150], [194], [196]

This framework can also explain material priorities under different etiologies. Post-traumatic OA usually requires addressing joint stability and abnormal load first. Materials mainly provide local shock absorption, load buffering, and sustained therapy. Age-related OA often has both matrix time-dependent mechanical changes and decreased intrinsic cellular adaptation. Therefore, dynamic mechanical environment and anti-aging metabolic regulation are more important. Obesity-related OA has both increased load and systemic metabolic inflammation. Mechanical protection and inflammatory metabolic intervention need to be considered together. Obvious subchondral bone sclerosis or local depression should place deeper osteochondral interface at higher priority. These relationships are not fixed rankings. They help material design shift from what material functions I have to what the current pathology most needs to correct.

Disease-modifying evaluation also needs to correspond to this phenotype stratification [200]. Traditional safranin O-fast green staining, type II collagen, aggrecan, and histological scoring remain important. However, they are insufficient to prove that mechanical pathology has been corrected. High-friction phenotypes require long-term friction and wear evaluation. Dynamic mechanical abnormality types require stress relaxation, fatigue, impact absorption, and gait evaluation. Mechanotransduction abnormality types require cellular mechanical thresholds and pathway causality validation. Inflammation-mechanics coupling types require simultaneous evaluation of mechanical input and synovial immune state. Osteochondral interface abnormality types require subchondral bone structure and cross-interface load transfer. Aging-metabolic types require joint evaluation of mitochondria, redox, senescence markers, and long-term mechanical tolerance. Functional endpoints are also indispensable [201]. Gait, load distribution, joint range of motion, and pain-related behavior can link microscopic material mechanical changes to overall joint use. For mechanobiology-instructed materials, these indicators are especially valuable (Fig. 10). True mechanical homeostasis recovery should manifest as the joint being able to bear load again during activity, not just as a more complete static tissue section.

**Fig. 10:**
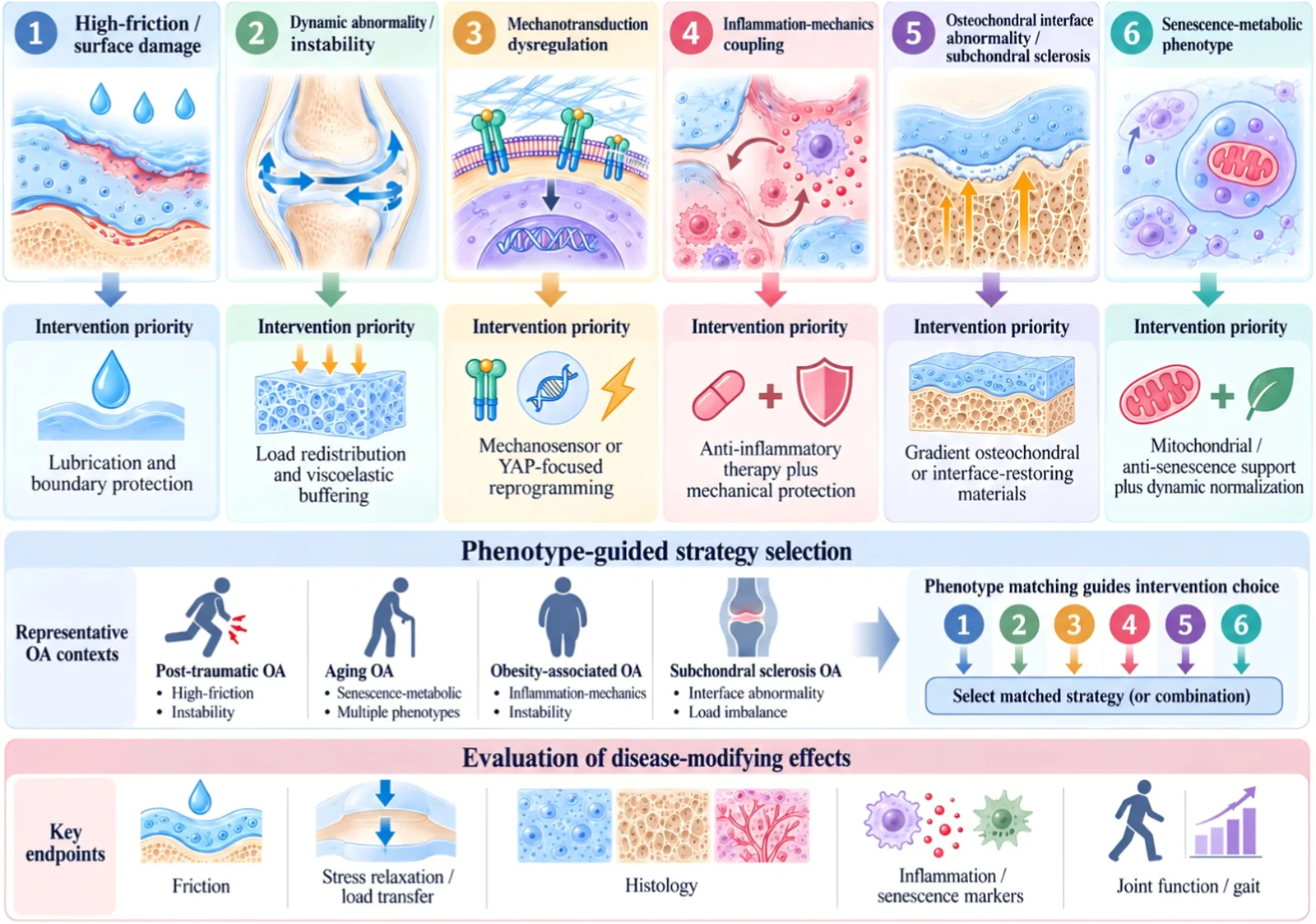
**Pathology-driven framework for disease-modifying evaluation of mechanobiology-instructed biomaterials.** Dominant mechanical pathology provides the basis for defining therapeutic priorities and matching material functions in heterogeneous OA. Disease modification requires mechanism-specific validation across material mechanics, cellular mechanotransduction, tissue responses, whole-joint function, and long-term therapeutic persistence.

Long-term persistence is another key dimension of disease modification. Systems dependent on ultrasound, electromagnetic stimulation, sustained drug release, or regular injection need to clarify whether efficacy is maintained after intervention stops. If mechanical sensitization, inflammation, or abnormal load rapidly return after stimulation stops, this is closer to a continuous treatment effect. If the material promotes stable reconstruction of tissue structure and mechanical environment and maintains lower disease progression after intervention stops, it is closer to true disease process modification. Therefore, disease-modifying evaluation of mechanobiology-instructed materials should form a continuous framework consisting of pathological phenotype identification, priority mechanical target determination, material function matching, cellular and tissue mechanism validation, whole-joint functional evaluation, and persistence observation after drug or stimulation withdrawal. Its core is not to specify a unique material for every OA. It is to give material selection a clear pathological basis. It also allows each material to be validated with mechanical and disease endpoints matching its design logic. Only at this level can lubrication, dynamic hydrogels, mechanoelectrical materials, mechanical-pharmacological systems, and osteochondral gradient structures no longer be parallel technology categories. They can be integrated as disease-modifying tools for different mechanical pathological phenotypes.

## 7 Conclusions and future perspectives

Mechanobiology-instructed biomaterials offer opportunities to move OA treatment beyond static mechanical matching toward the regulation of pathological mechanical information. However, increasingly sophisticated material designs do not necessarily translate into effective disease modification. The key challenge is to establish reliable connections among disease-specific mechanical abnormalities, material-mediated mechanical regulation, cellular mechanotransduction, and sustained whole-joint outcomes. Addressing the following challenges will be essential for translating these conceptual advances into clinically applicable therapeutic strategies.

### 7.1 Beyond static modulus and toward clinically relevant mechanical characterization

Compressive modulus remains a commonly used parameter for cartilage biomaterials, yet the mechanical microenvironment experienced by cells also depends on viscoelasticity, stress relaxation, permeability, interfacial friction, and cyclic fatigue. Similar initial stiffness does not necessarily produce comparable cellular mechanical responses. Future material characterization should therefore incorporate physiologically relevant dynamic loading and evaluate changes in mechanical properties during repeated use and degradation. Such measurements would provide a more biologically meaningful basis for material design and therapeutic evaluation. An additional translational challenge is the lack of validated biomarkers and standardized criteria for identifying dominant mechanical phenotypes in individual patients. Although imaging, biomechanical assessments, and molecular biomarkers may provide complementary information, their ability to guide phenotype-specific material selection remains insufficiently established. Integrating these measurements with longitudinal clinical outcomes and therapeutic responses may help establish more reliable strategies for patient stratification and personalized material design.

### 7.2 From mechanotransduction association to causal validation

Although changes in mechanotransduction-related pathways, including Piezo1, FAK, and YAP/TAZ, are frequently reported following biomaterial intervention, such associations do not necessarily establish a causal relationship between material mechanics and therapeutic outcomes. Mechanical effects may also overlap with those arising from material chemistry, drug release, or secondary inflammatory responses. Future investigations should therefore combine controlled mechanical parameter variation with direct measurements of cellular deformation, traction forces, or local strain. Genetic and pharmacological inhibition, together with rescue experiments, may further clarify the contribution of specific mechanotransduction pathways. Establishing causal relationships among material properties, local mechanical cues, cellular responses, and disease-related outcomes will be critical for distinguishing mechanobiological regulation from general biomaterial-mediated therapeutic effects.

### 7.3 Mechanics–pharmacology coupling for disease modification

Mechanical abnormalities in OA frequently coexist with inflammation, oxidative stress, metabolic dysfunction, and cellular senescence. Mechanical regulation alone may be insufficient to reverse established pathological cell states, while pharmacological treatment cannot fully compensate for persistent abnormal loading or friction. Mechanism-guided integration of mechanical regulation and pharmacological intervention may therefore provide complementary therapeutic benefits. Future studies should determine whether mechanical protection can improve therapeutic responsiveness, reduce the required drug dose, or restore cellular adaptation to physiological mechanical stimuli. Equally important is distinguishing genuine therapeutic synergy from the additive effects of independently functioning components. Such evidence would help establish the rationale for integrating mechanical and pharmacological functions in disease-modifying biomaterials.

### 7.4 Toward durable whole-joint homeostasis and clinical translation

The ultimate goal of mechanobiology-instructed biomaterials is not simply to restore a particular mechanical parameter, but to establish a mechanical environment capable of supporting sustained joint homeostasis. Future evaluation should extend beyond local cartilage histology to integrate interfacial friction, dynamic tissue mechanics, gait and load-bearing function, synovial inflammation, and subchondral bone remodeling. These measurements should be assessed under physiologically relevant loading conditions and over sufficiently long observation periods to determine whether improvements in tissue structure are accompanied by meaningful functional recovery. A particularly important unresolved question concerns the persistence of mechanical regulation during and after material degradation. Changes in stiffness, viscoelasticity, lubrication, and load-transfer properties during degradation may alter the mechanical signals transmitted to surrounding cells. Whether the restored mechanical microenvironment can be maintained through stable host tissue remodeling after the material has been resorbed remains uncertain. For drug-delivery or externally stimulated systems, therapeutic durability after treatment withdrawal also requires validation. These considerations highlight the importance of distinguishing sustained disease modification from temporary mechanical protection or symptomatic improvement. Beyond mechanistic and functional validation, clinical translation will require attention to manufacturing reproducibility, material delivery, long-term biocompatibility, and the feasibility of integrating biomaterial interventions into existing treatment strategies. Prospective studies linking patient-specific mechanical characteristics with therapeutic responses will be particularly important for determining the clinical utility of phenotype-guided material selection. Progress in these areas may help establish a more practical pathway from mechanobiological understanding to durable, clinically meaningful OA intervention.

Overall, future advances will depend on improving the biological relevance of mechanical characterization, establishing causal mechanotransduction mechanisms, and demonstrating long-term functional benefits in clinically relevant settings. Addressing these challenges may help bridge the gap between mechanotransduction research and the development of biomaterials with genuine disease-modifying potential.

## CRediT authorship contribution statement

**Jingxin Pan:** Writing – original draft, Visualization, Resources, Investigation, Formal analysis, Data curation, Conceptualization. **Xi Wang:** Software, Investigation, Formal analysis, Data curation, Conceptualization. **Pan Li:** Resources, Data curation, Conceptualization. **Jianbing Ma:** Writing – review & editing, Writing – original draft, Validation, Formal analysis, Data curation, Conceptualization. **Zhiyuan Wang:** Writing – review & editing, Project administration, Investigation, Formal analysis, Data curation, Conceptualization, Funding acquisition, Writing – original draft, Supervision, Validation. **Zhao Zhang:** Writing – review & editing, Writing – original draft, Validation, Supervision, Project administration, Investigation, Funding acquisition, Formal analysis, Data curation, Conceptualization.

## Funding

This work was supported by the 10.13039/501100001809National Natural Science Foundation of China (Grant No. 82401852), 10.13039/501100007128Natural Science Foundation of Shaanxi Province (No. 2021JQ-927), the 18th Special Funding of the 10.13039/501100002858China Postdoctoral Science Foundation (No.2025T181192) and the Young Talent Support Program of Shaanxi Association for Science and Technology (Grant No. 20250346)

## Declaration of competing interest

The authors declare that they have no known competing financial interests or personal relationships that could have appeared to influence the work reported in this paper.

## Data Availability

No data was used for the research described in the article.

## **Abbreviations**

AKT: protein kinase B
DNA: deoxyribonucleic acid
ECM: extracellular matrix
FAK: focal adhesion kinase
FEA: finite element analysis
FOXC1: forkhead box C1
HDAC6: histone deacetylase 6
IL,: interleukin
JAK2: Janus kinase 2
NF-κB: nuclear factor kappa B
OA: osteoarthritis
OTUD4: OTU deubiquitinase 4
PDZK1: PDZ domain-containing protein 1
PI3K: phosphoinositide 3-kinase
PTOA: post-traumatic osteoarthritis
RhoA: Ras homolog family member A
ROCK: Rho-associated coiled-coil-containing protein kinase
ROS: reactive oxygen species
SEM: scanning electron microscopy
SOX9: SRY-box transcription factor 9
STAT3: signal transducer and activator of transcription 3
STING: stimulator of interferon genes
TRPV4: transient receptor potential vanilloid 4
TXNIP: thioredoxin-interacting protein
YAP: Yes-associated protein
TAZ: transcriptional coactivator with PDZ-binding motif
